# Reconstructing Roma History from Genome-Wide Data

**DOI:** 10.1371/journal.pone.0058633

**Published:** 2013-03-13

**Authors:** Priya Moorjani, Nick Patterson, Po-Ru Loh, Mark Lipson, Péter Kisfali, Bela I. Melegh, Michael Bonin, Ľudevít Kádaši, Olaf Rieß, Bonnie Berger, David Reich, Béla Melegh

**Affiliations:** 1 Department of Genetics, Harvard Medical School, Boston, Massachusetts, United States of America; 2 Program in Medical and Population Genetics, Broad Institute, Cambridge, Massachusetts, United States of America; 3 Department of Mathematics and Computer Science and Artificial Intelligence Laboratory, Massachusetts Institute of Technology, Cambridge, Massachusetts, United States of America; 4 Department of Medical Genetics and Szentagothai Research Center, University of Pécs, Pécs, Hungary; 5 Department of Medical Genetics, University of Tübingen, Tübingen, Germany; 6 Institute of Molecular Physiology and Genetics, Slovak Academy of Sciences, Bratislava, Slovakia; University of Oxford, United Kingdom

## Abstract

The Roma people, living throughout Europe and West Asia, are a diverse population linked by the Romani language and culture. Previous linguistic and genetic studies have suggested that the Roma migrated into Europe from South Asia about 1,000–1,500 years ago. Genetic inferences about Roma history have mostly focused on the Y chromosome and mitochondrial DNA. To explore what additional information can be learned from genome-wide data, we analyzed data from six Roma groups that we genotyped at hundreds of thousands of single nucleotide polymorphisms (SNPs). We estimate that the Roma harbor about 80% West Eurasian ancestry–derived from a combination of European and South Asian sources–and that the date of admixture of South Asian and European ancestry was about 850 years before present. We provide evidence for Eastern Europe being a major source of European ancestry, and North-west India being a major source of the South Asian ancestry in the Roma. By computing allele sharing as a measure of linkage disequilibrium, we estimate that the migration of Roma out of the Indian subcontinent was accompanied by a severe founder event, which appears to have been followed by a major demographic expansion after the arrival in Europe.

## Introduction

The Roma (also called Romani) are a unique and diverse population that live in Europe, Near East, Caucasus, and the Americas. They speak more than 60 dialects of a rapidly evolving language called *Romani* and belong to various social and religious groups across Europe. Their census size has been estimated to be in the range of 10–15 million [1], with the largest populations in Eastern Europe [2]. They do not have written history or genealogy (as Romani does not have a single convention for writing) and thus most of the information about their history has been inferred based on linguistics, genetics and historical records of the countries where they have resided.

Historical studies have suggested that the Roma are originally from India, and that they migrated to Europe between the 5^th^ and 10^th^ century [3]. It has been argued that their migration route included Persia, Armenia, Anatolia, and Greece [3], [4]. The Roma then settled in multiple locations within Europe and were widespread in Europe by the 15^th^ century; descendents of these migrants currently live primarily in the Balkans, Spain, and Portugal [5].

Anthropological and linguistic studies have documented striking similarities between the cultures and languages of various Indian groups and Roma. Social structure in Roma groups is similar to the *castes* of India, where the groups are often defined by profession [2], [3]. Like many Indian populations, the Roma practice endogamy and individuals of one Roma clan (sub-ethnic group) preferentially marry within the same group, and marriages across clans are proscribed [3]. Anthropological studies have also suggested a link between the Roma and Banjara (nomadic gypsy groups) residing in India [3] (even though linguistic analysis of the *Banjari* or *Lamani*, languages spoken by the Indian nomadic groups, have little similarity to Romani [6]). Comparative linguistics have further suggested that Northwestern Indian languages like Punjabi or Kashmiri or Central Indian languages like Hindi are most closely related to Romani [9], [10].

Genetics provides a complementary source of information to data from history, archaeology and linguistics. Y-chromosome marker H1a-M82 and mitochondrial haplogroups M5a1, M18 and M35b that are thought to be characteristic of South Asian ancestry, are present at high frequency in Roma populations [7], [8]. However, there is no consensus about the specific ancestral group/geographic region within South Asia that is most closely related to the ancestral population of the Roma. A recent study based on Y-chromosome markers showed that the Roma descended from southern Indian groups [11], which contradicts previous reports based on mtDNA haplogroups that have placed the origin of Roma in Northwest India. While mtDNA and Y chromosome analyses provide valuable information about the maternal and paternal lineages, a limitation of these studies is that they represent only one instantiation of the genealogical process. Autosomal data permits simultaneous analysis of multiple lineages, which can provide novel information about population history.

Here we analyze whole genome SNP array data from 27 Roma samples belonging to six groups sampled from 4 countries in Europe (three separate ethnic groups from Hungary, and one group each from Romania, Spain and Slovakia). Our aim was to address the following questions: (1) What is the source of the European ancestry in the Roma? (2) What is the relationship of the Roma to the present-day South Asian populations? (3) What is the proportion and timing of major gene flow into this population? (4) Can we characterize the founder events that have occurred in the history of this population?

## Results

### Genome-wide Ancestry Analysis of the Roma

**Figure 1:**
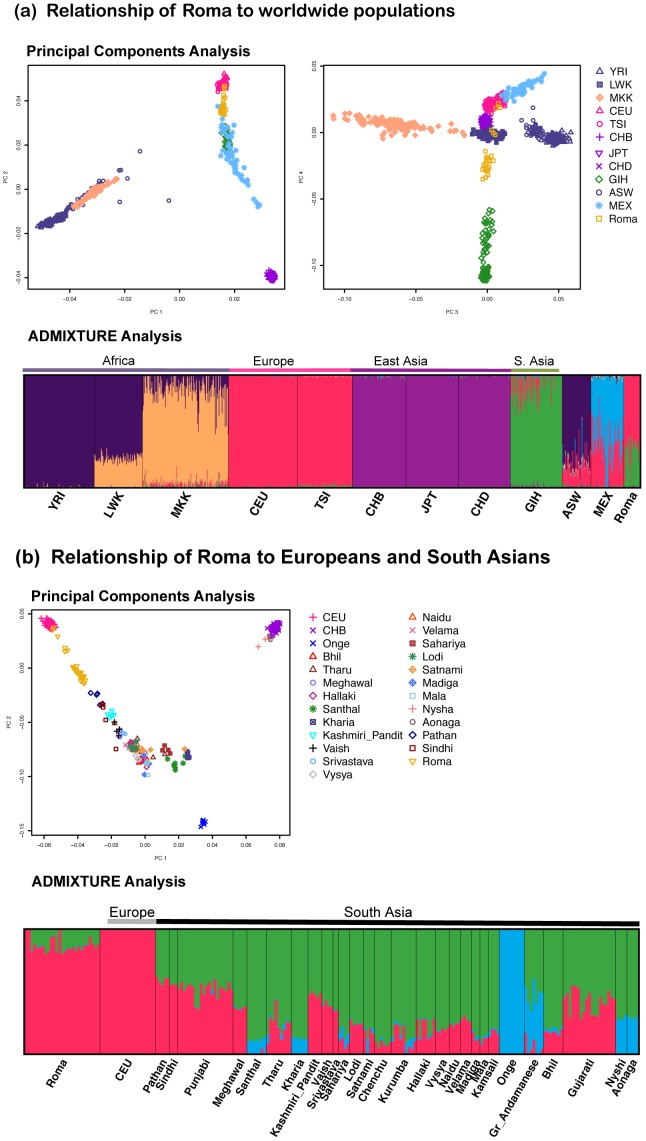
Relationship of Roma with other worldwide populations. We applied PCA and ADMIXTURE to study the relationship of Roma with the HapMap and South Asian populations. In PCA, each point represents an individual, and in ADMIXTURE, each line represents an individual. (a) shows the PCA and ADMIXTURE results for clustering of Roma and HapMap populations. The populations codes are as follows: Yoruba in Ibadan, Nigeria (YRI), Luhya in Webuye, Kenya (LWK), Maasai in Kinyawa, Kenya (MKK), Utah residents with Northern and Western European ancestry (CEU), Toscani in Italia (TSI), Han Chinese in Beijing, China (CHB), Japanese in Tokyo, Japan (JPT), Chinese in Metropolitan Denver, Colorado (CHD), Gujarati Indians in Houston, Texas (GIH), African ancestry in Southwest USA (ASW) and Mexican ancestry in Los Angeles, California (MEX), and (b) shows the PCA and ADMIXTURE results for clustering of Roma and South Asian groups. We limit the sample size of all groups (except Roma) to 20 individuals.

We applied Principal Component Analysis (PCA) using the SMARTPCA software [12] and the clustering algorithm ADMIXTURE [13] to study the relationship of Roma to other worldwide populations in a merged dataset of Roma and HapMap populations. In PCA, the Roma fall between the South Asians (Gujaratis) and Europeans, consistent with Roma deriving ancestry both South Asians and Europeans and in line with previous mtDNA and Y chromosome analyses [7], [8] (Figure 1). The ADMIXTURE software, which implements a maximum likelihood method to infer the genetic ancestry of each individual modeled as a mixture of K ancestral groups, produces very similar inferences [13]. At K = 6 (which has the lowest cross-validation error), we observe clustering based on major continental ancestry. Similar to the PCA results, the Roma individuals cluster with South Asians and Europeans (Figure 1, Figure S1). We also examined pairwise average allele frequency differentiation (F_st_) between Roma and major continental groups (see Table S1) and observed that they have the lowest F_st_ with other European groups.

Previous studies have shown that the HapMap Gujarati population is not an ideal surrogate for the variation in India, as this group is heterogeneous and has recent West Eurasian ancestry [14]. To study the relationship of Roma to South Asians, we repeated the clustering analysis with Roma, Europeans and 28 South Asian groups (24 Indian groups from the India Project (we remove Siddis as they have recent African ancestry), Pathan and Sindhi from HGDP and Punjabi and Gujarati from POPRES). As previously seen in PCA, we observed that all Indians fall on a cline of variable relatedness to Europeans and indigenous Andamanese population (Onge) [14]. The Roma also fall on this cline but they appear to be closest to the European cluster compared to any other South Asian group included (Figure 1b). Similar results were observed in our ADMIXTURE analysis (Figure 1b, Figure S1). Based on the PCA and ADMIXTURE analysis, we excluded three Roma outlier samples from further analyses, as they appeared to have very recent admixture from neighboring non-Roma European populations (likely in the past few generations).

We applied the *4 Population Test* [*14*] to formally examine if the Roma have evidence of a mixture of European and South Asian ancestry. We used individuals of Northern European ancestry (CEU) and Andamanese (Onge) as surrogates for the European and South Asian ancestral populations respectively. We tested whether the phylogenetic tree (Africans, Europeans, South Asians, Roma) is consistent with the data. We choose Onge for this analysis, since, unlike their distant relatives on the Indian mainland, they do not have any evidence of West Eurasian related admixture [14]. Applying the *4 Population Test*, we observed highly significant violations of the expected phylogenetic tree topology, confirming that the Roma are admixed; that is, they have ancestry from both South Asians and Europeans (Table S2). We note that this test does not distinguish between European and West Asian ancestry and qualitatively similar results would be observed if we replace CEU with any other West Eurasian population (other groups from Europe, Middle East, Central Asia or Caucasus), hence we refer to this ancestry component as “Ancestral West Eurasian (AWE)”.

To quantify the magnitude of the South Asian and West Eurasian ancestry in the Roma, we applied *F_4_ Ratio Estimation* [*15*] using the model shown in Figure S2, which can estimate admixture proportions in the absence of data from good surrogates of the ancestral populations. Here, we used CEU and Adygei (a population from the Caucasus) represent the West Eurasian component and Onge to represent the ancestral South Asian component (referred to as Ancestral South Indian (ASI)) as they do not have any West Eurasian ancestry [14]. The *F_4_ Ratio Estimation* is known to work only if we have access to data from populations that form a clade with the unadmixed ancestral populations. Since all populations in mainland India are admixed none are appropriate for this test [14]. To further evaluate our model of population relationships in Figure S2, we used *admixture graph* [*15*] and found that this model provides a good fit to the data.

Applying the *F_4_ Ratio Estimation* to Roma (pooling all samples together), we estimate that the Roma have on average 77.5±1.8% West Eurasian related ancestry (standard errors were computed using a Block Jackknife with a block size of 5 cM) (Table S2). As all Indian groups harbor ancestry from a West Eurasian related populations (previously referred to as Ancestral North Indian (ANI) ancestry [14]), we note that some of West Eurasian related ancestry we detect in Roma likely derives from India itself–from the ANI–while other parts may be from European or Middle Eastern admixture (post exodus from India).

### Estimating a Date of European Admixture in the Roma

To infer the date of the gene flow, we applied a modified version of *ROLLOFF* [*16*], which uses the decay of admixture linkage disequilibrium (LD) to estimate the time of admixture. *ROLLOFF* computes SNP correlations in the admixed population and weights the correlations by the allele frequency difference in the ancestral populations such that the signal is sensitive to admixture LD. While this method estimates accurate dates of admixture in most cases, we observed that it is noticeably biased in case of strong founder events post admixture (Table S3). The bias is related to a normalization term that exhibits an exponential decay behavior in the presence of a strong founder event, thus confounding the admixture date (see details in Note S1, Figure S3). We propose a modification to the *ROLLOFF* statistic that removes the bias (Note S1, Table S3). In addition, the new statistic computes covariance instead of correlation between SNPs; this does not affect the performance of the method but makes it mathematically more tractable. Throughout the manuscript, we use the modified *ROLLOFF* statistic (*R*(*d*)) unless specified otherwise. Simulations show that this statistic gives accurate and unbiased results up to 300 generations (Note S2, Figure S4).

A feature of *ROLLOFF* is that it uses allele frequency information in the ancestral populations to amplify the admixture signal relative to background LD. While data from the ancestral populations is not available for Roma, this information can be obtained by performing PCA using present day Europeans and South Asians. Simulations show that using PCA-based SNP loading effectively captures the allele frequency differentiation between the ancestral populations and can be used for estimating dates of mixture (Note S2, Figure S5).

**Figure 2:**
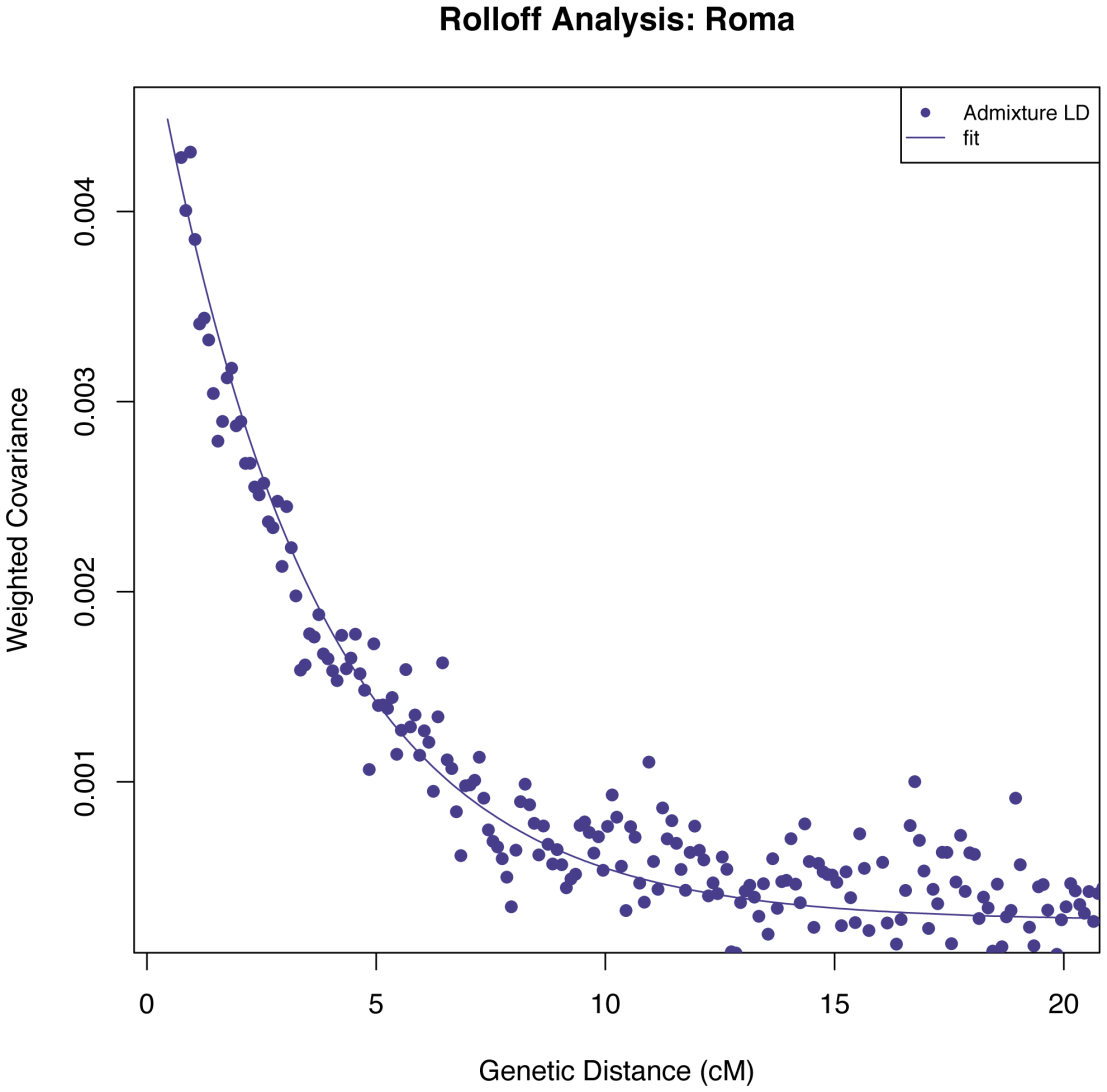
Admixture date estimation. We performed *ROLLOFF* (using *R(d)*) on the Roma samples (*n* = 24). We plot the weighted covariance as a function of genetic distance, and obtain a date by fitting an exponential function with an affine term: 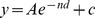, where *d* is the genetic distance in Morgans and *n* is the number of generations since mixture. We do not show inter-SNP intervals of <0.5 cM since we have found that at this distance admixture LD begins to be confounded by background LD.

Applying the *ROLLOFF* (using R(d)) to the Roma samples with the SNP loading estimated using PCA of Europeans (CEU) and 16 Indian groups (limited to groups that fall on the main cline of West Eurasian relatedness in PCA so that the signal is not confounded by other ancestry components), we estimate that the West Eurasian admixture in Roma occurred 29±2 generations or about 780–900 years ago, assuming one generation = 29 years [17] (Figure 2). This is consistent with mixture having occurred only after the historically recorded arrival of the Roma in Europe between 1,000–1,500 years ago [3].

A potential complication is that the date we are estimating may also be reflecting earlier admixture with ANI in India and any gene flow from Middle Eastern populations that occurred after the Roma exodus from India. The allele frequency of ANI and Middle Eastern populations are correlated to the allele frequencies of the Europeans used in the analysis, and hence the date of admixture inferred using a single exponential function should be interpreted as an average date of all West Eurasian related gene flow events. When we consider a two-pulse model of admixture (by fitting a sum of two exponential functions to infer the dates), we obtain dates of 37 and 4 generations. The older date corresponds to about 1,000 years before present – again consistent with the historical record – and both dates are much more recent than any estimates obtained by applying *ROLLOFF* in India. This suggests that the admixture we are detecting is genuinely related to events that occurred after the exodus from India.

### Source of the European Ancestry in Roma

**Figure 3:**
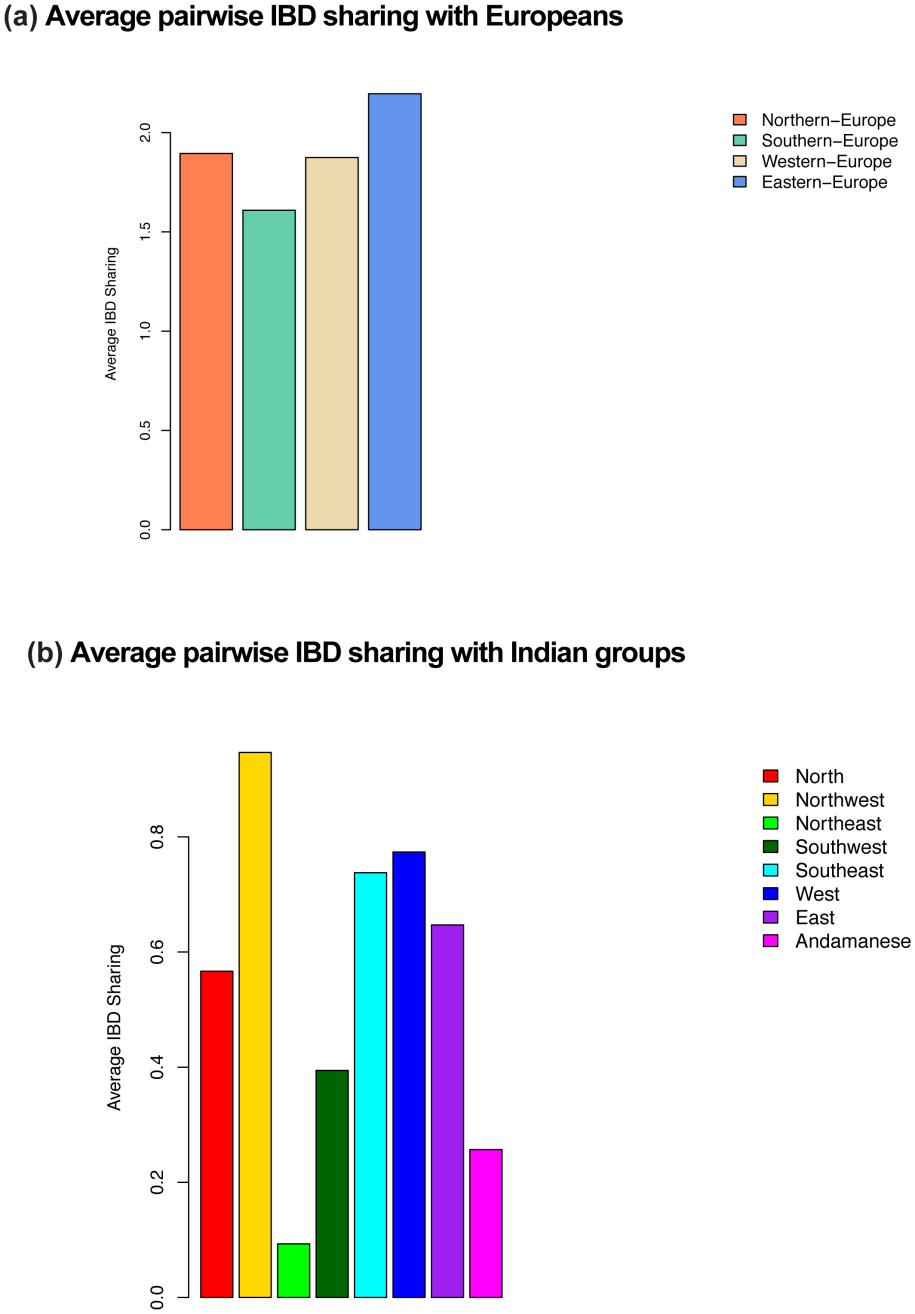
The European and South Asian sources of Roma ancestry. We computed a genome-wide average IBD sharing distance between Roma (all samples combined in one group) and other regional groups. Details of the regional grouping are described in Methods. (a) shows the average pairwise IBD sharing between Roma and Europeans (grouped into four regional categories), (b) shows IBD sharing average pairwise IBD sharing between Roma and South Asians (grouped into 8 regional categories).

To learn about the relationship of the Roma to European populations, we estimated the pairwise Identity-by-descent (IBD) sharing between each Roma individual and non-Roma European individual. We grouped the European samples from POPRES, HapMap and HGDP into four major regional groups: Northern (*n* = 595), Southern (*n* = 649), Eastern (*n* = 82), and Western Europe (*n* = 241). IBD segments (>3 centimorgans (cM)) were detected using GERMLINE [18]. Next, we computed an average pairwise sharing distance between Roma and the European groups in each region (see Methods). We observed that Roma exhibit the highest IBD sharing with individuals from Eastern Europe (Figure 3a). When we perform stratified analysis (where Roma individuals from each country were considered separately), we observed that the highest sharing for each Roma group is still with Eastern Europeans (even for Roma individuals from Spain) (Figure S6).

### Source of the South Asian Ancestry in Roma

To learn about the source of the South Asian ancestry in Roma, we inferred the pairwise IBD sharing distance between Roma and various South Asian groups. Again, we performed GERMLINE analysis to compute the average pairwise sharing distance between Roma and 28 South Asian populations (from India Project, HGDP and POPRES). To simplify the analysis, we classified the samples into 8 groups based on geographical region within India: North (*n* = 38), Northwest (*n* = 225), Northeast (*n* = 8), Southwest (*n* = 16), Southeast (*n* = 29), East (*n* = 11), West (*n* = 32), and Andamanese (*n* = 16). We observe that the Roma share the highest proportion of IBD segments with groups from the Northwest of India (Figure 3b). Interestingly, the two Northwest Indian groups that show the highest relatedness to Roma (Punjabi, Kashmiri Pandit) are also the populations that have highest proportion of West Eurasian-related (ANI) ancestry in our sample. To control for the possibility that the high IBD sharing could be an artifact related to high ANI ancestry, we recalculated the IBD sharing regressing out the ANI ancestry proportion and observed that the Roma continue to share the highest IBD segments with the Northwest Indian groups (Note S3). These findings are consistent with analyses of mtDNA that also place the most likely South Asian source of the Roma in Northwest India [8].

An important caveat is that we have large variation in the number of samples from each regional group, with some groups containing only a handful of samples. In order to control for the sample sizes, we performed bootstrap analysis drawing a random sample of up to 30 individuals from each regional group and recomputed the IBD statistics. We repeated the process 100 times and estimated the mean and standard error (Note S3). We observed that Roma continue to share the highest IBD segments with Northwest Indian groups. There is very little variability across the 100 runs, suggesting that this analysis may also be picking up founder events shared between Roma and Indian groups (Note S3, Figure S7).

### Characterizing the Founder Events

**Figure 4:**
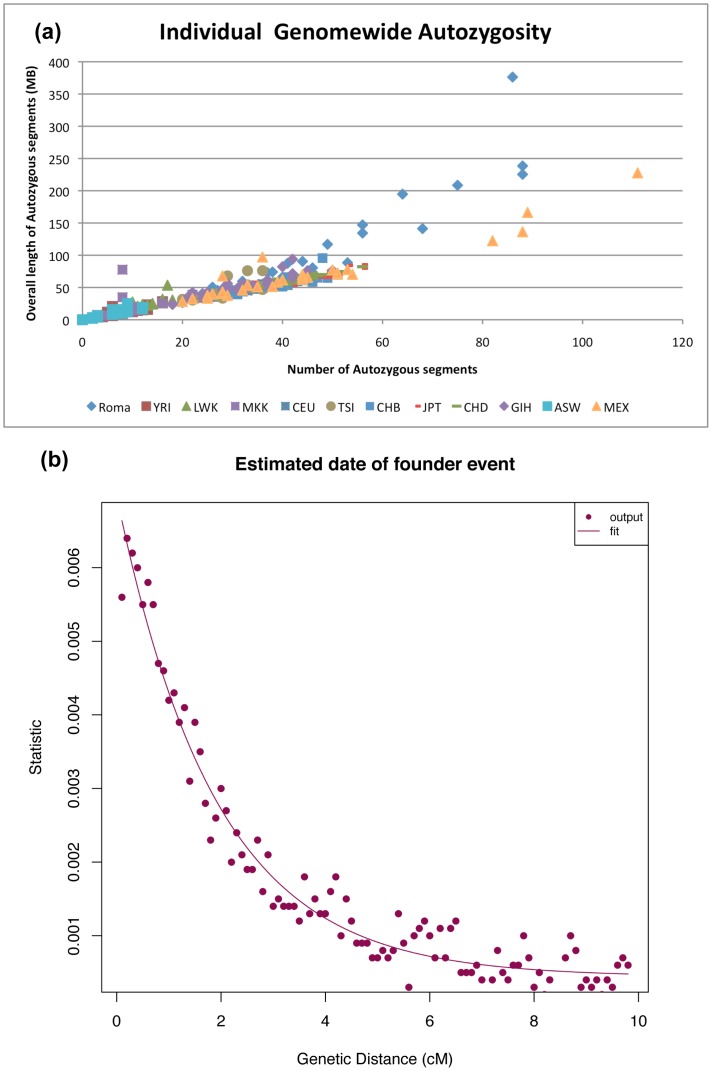
Founder events in the Roma. (a) shows estimates of genomewide autozygosity in Roma and individuals from HapMap (*n* = 30 from each of the 11 HapMap populations). Each point represents an individual with the color-coding described in the legend. (b) shows the decay of autocorrelation with genetic distance. We fit an exponential function: 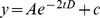 where *D* = distance in Morgans and *t* = time of founder event. We thus infer a founder event date of 27 generations.

Previous genetic and social studies have shown that the present day Roma population has descended from a small number of ancestors with subsequent genetic and cultural isolation [8], [19]. A history of founder events in a population can lead to an increase in homozygosity and large stretches of allele sharing across individuals within the same population. This can be measured by estimating the proportion of the autosomal genome that has homozygous genotypes. We applied PLINK v1.07 [20] to compute a genomic measure of individual autozygosity for all Roma individuals and 30 random individuals from each of the 11 HapMap populations. PLINK uses a sliding window approach to find regions of the genome that are at least 1 MB in length and contain 100 contiguous homozygous SNPs. For each individual, we computed the number and overall length of the autozygous segments and observed that the Roma have very high levels of autozygosity compared to other HapMap populations (Figure 4a). This suggests that inbreeding (or consanguineous marriages) might be common in Roma.

To infer the date of the founder event in Roma, we studied the relationship of allele sharing with increasing distance as reported in Reich et al (2009) [21]. This statistic is based on examining the autocorrelation of allele sharing between pairs of individuals within a population, and then subtracting the cross-population autocorrelation to remove the effects of ancestral allele sharing inherited from the common ancestor. By measuring the exponential decay of auto-correlation with genetic distance, we obtained an estimate of the age of the founder event. Simulations have shown that this method can accurately estimate the dates of recent founder events, even in admixed populations (Note S4, Table S5).

Applying this method to Roma and subtracting the shared Roma and European (CEU) autocorrelation, we estimate that a Roma founder event occurred 27 generations or ∼800 years ago (assuming one generation = 29 years [17]) (Figure 4b). This is consistent with reports that the Roma exodus from India occurred 1,000 years ago [3], and suggests that the migration out of the Indian sub-continent may have been associated with a significant founder event in which a small number of ancestral individuals gave rise to the present-day Roma population.

## Discussion

Using genome-wide SNP data from Roma individuals, we have provided (1) confirmation of previous mtDNA and Y chromosome results with autosomal data, and (2) some new insights that take special advantage of autosomal data.

We have performed formal tests to confirm that Roma are admixed and have ancestry from two highly divergent populations: a West Eurasian population and a South Asian population. We estimate that the Roma have 77.5% West Eurasian ancestry, reflecting a combined estimate of the ANI ancestry that the Roma derive from their South Asian ancestors (pre-exodus) and the European ancestry related to the admixture in Europe (post-exodus from India). Our estimate of West Eurasian ancestry is broadly consistent with admixture proportions estimated using autosomal short tandem repeats (66–100%) [22]. Our estimates of non-West Eurasian ancestry (ASI = 22.5±1.8%) are also consistent with the estimates from mitochondrial DNA (26.5%) and Y-chromosome (16.7%) markers [23], [24].

Our identity-by-descent analysis provides novel insights related to the source of the ancestral populations of Roma. We provide evidence for Eastern Europe being a major source of the European ancestry, and Northwest India being a major source of the South Asian ancestry in Roma. Our inferences about the geographic origin within South Asia help resolve a long-standing debate related to the origin of the Romani people. Our results are consistent with reports from linguistics [9] and mtDNA studies [8], which have shown that present day Northwest Indian populations (from Kashmir and Punjab), are candidates for being the source of the Indian ancestry in Roma [8], [23]. However, we caution that IBD based methods require large sample sizes to be well powered to detect subtle differences between geographic regions.

A historically informative insight from our analysis is the date of the West Eurasian gene flow into Roma. Using a statistic that captures the pattern of admixture related linkage disequilibrium; we estimate that the admixture between Roma and West Eurasians occurred 29±2 generations or about 780–900 years ago. The earliest records of the arrival of Roma in the Balkans dates back to the 11^th^–12^th^ century [3], which is concordant with our estimated date of mixture [3]. It is important to note that the Roma have ancestry from both ANI and Europeans and thus the estimated date of admixture with Europeans (post exodus) is slightly downward biased (older). Simulations have shown in the case of two gene flow events, the date of admixture estimated by *ROLLOFF* tends to reflect the date of the more recent gene flow event as the interval between the dates of two gene flow events increases (Table S4, Note S2).

Disease mutation screening in the Roma has shown that they have an increased proportion of private mutations [19]. For example, deletion 1267delG is known to cause a neuromuscular disorder, *congenital myasthenia*, and has a high carrier frequency in many Roma groups that reside in different parts of Europe. This mutation has only been observed in South Asian populations previously [19], [25]. This provides evidence that the different Roma groups have a history of a shared founder events with South Asians. In order to obtain temporal information of the founder event that has likely increased the frequency of such disease causing mutations in Roma, we studied LD based allele sharing statistics and estimated that the founder event in Roma occurred about 27 generations, or 800 years, ago. This agrees with previous reports from Morar et al. (2004) [25] who hypothesize that the entire Roma population was founded about 32–40 generations ago.

After this manuscript was submitted, two other studies characterizing the population history of Roma were published. First, a study based on Y-chromosome haplogroups showed that on the paternal lineage, Roma haplotypes cluster predominantly with the Northwestern Indian haplotypes [26], consistent with our findings based on autosomal IBD sharing. The second study was based on whole genome SNP genotype data like ours [27]. Our findings are broadly consistent with the results from that paper, although with some notable differences. For inferring the date of the founder event, the other study uses a two-pulse model (an out-of-India founder event, followed by a second founder event that affects only the western Roma groups). We instead estimate the date of a single shared founder event; with our limited sample size (we have only 2 samples from western Roma groups), we cannot recover the entire distribution of founder events and so the date of the founder event in our study should be interpreted as an average date of multiple founder events. Similarly, the other study, using a continuous admixture model, estimates that the admixture in Roma occurred over a period of 38 generations [27]. Assuming a single admixture model, we estimate that the average date of admixture is 29±2 generations. However, when we consider a two-pulse model of admixture, we infer the dates of 37 and 4 generations, consistent with the results of the other study.

In conclusion, our study has confirmed that the Roma have ancestry from South Asians (likely Northwest Indians) and West Eurasians (likely Eastern Europeans), with mixture occurring around 30 generations ago and major founder events shortly afterward. An important opportunity for future work is to perform homozygosity mapping in Roma that can aid in finding disease-causing mutations related to the founder events.

## Materials and Methods

### Datasets

We collected 27 Roma samples belonging to six groups that were sampled from four countries in Europe from Hungary (3 linguistically and culturally separated sub-groups: 7 samples from Olah (Vlah), 4 samples from Beas (Boyash) and 4 samples from Romungro), 4 samples from Romania, 4 samples from Spain and 4 samples from Slovakia (Slovakian speaking Roma). All research involving human participants was approved by the Regional Ethics Committee Board (REKEB) and the Hungarian National Ethics Committee (ETT TUKEB). Each study participant attended a 45–60 mins verbal orientation session about the study design and goals and then provided written informed consent. All the research was conducted according to the principles expressed in the Declaration of Helsinki. Roma individuals self-reported as being descendants of the same tribe for at least three generations. The samples were genotyped using an Affymetrix 1 M SNP chip. We required <5% missing genotypes per sample per SNP to be included in the analysis (27 individuals, 726,404 SNPs passed this threshold). These data were merged with data from four other sources, including the International Haplotype Map Phase 3 (HapMap) (*n* = 1,115 samples from 11 populations genotyped on Affymetrix 1 M array) [28], the CEPH-Human Genome Diversity Panel (HGDP) (*n* = 257 individuals from 51 populations genotyped on Affymetrix 500 K SNP array) [29], [30], our previous study of Indian genetic variation which we call the “India Project” in this paper (*n* = 132 individuals from 25 groups genotyped on an Affymetrix 1 M SNP array) [14] and the Population Reference Sample (POPRES) (*n* = 3,845 individuals from 37 European populations genotyped on an Affymetrix 500 K SNP array) [31]. Depending on the analyses, we included different number of reference populations from these sources.

### Population Structure Analysis and F_st_ Calculation

To study the relationship of Roma with HapMap populations, we created a merged dataset of Roma and HapMap populations (*n* = 1,142 and 726,404 SNPs). As background LD can affect both PCA and ADMIXTURE analysis, we thinned the marker set using PLINK v1.07 [20] by excluding SNPs in strong LD (pairwise genotypic correlation r^2^>0.1) in a window of 50 SNPs (sliding the window by 5 SNPs at a time). The thinned dataset contained 61,052 SNPs. We used SMARTPCA [12] to perform PCA and to compute F_ST_ values. Clustering analysis was performed using ADMIXTURE [13].

To study the relationship of Roma with South Asians, we created a merged dataset of Roma, HapMap, POPRES and HGDP (*n* = 1,966 and 205,710 SNPs) and performed PCA and ADMIXTURE using the LD thinned dataset containing 55,303 SNPs.

### Formal Tests of Population Mixture

To test if Roma have West Eurasian and Indian ancestry, we used the unrooted phylogenetic tree ((YRI, CEU), (Onge, Roma)) and computed the *4-population test* statistic for all three phylogenetic trees that can possibly relate these populations. For this analysis, we created a merged dataset of Roma, India project and HapMap populations (*n* = 1,274 and 524,053 SNPs). Let YRI_i_, CEU_i_, Onge_i_ and Roma_i_ be the allele frequencies for SNP *i* in the populations YRI, CEU, Onge and Roma respectively. Specifically, we compute the correlation: *ρ*(YRI_i_-CEU_i_, Onge_i_-Roma_i_) for all SNPs across the genome. In the absence of mixture, the expected correlation would be 0. Standard errors were computed using Block Jackknife [32], [33] where a block of 5 cM was dropped in each run.

### Estimating Genome-wide Ancestry Proportion

We estimate the genome-wide proportion of ancestry using *F_4_ Ratio Estimation* [*15*] which estimates the excess of West Eurasian ancestry compared to Onge. We use the model of population relationships shown in the Figure S2. We test this model using admixture graph [15] and find that the model is a good fit to the data (meaning that none of the f-statistics are greater than three standard errors from expectation). *F_4_ Ratio Estimation* computes the ratio of *f_4_*(YRI_i_, Adygei_i_; Roma_i_-Onge_i_)/*f_4_*(YRI_i_, Adygei_i_; CEU_i_-Onge_i_). This quantity is summed over all sites (262,558 SNPs) and the standard errors are computed using the Block Jackknife (block size of 5 cM). To represent all the populations needed for this analysis, we created a merged dataset that included data from the Roma, the India project, HGDP and HapMap (*n* = 1,531 and 262,558 SNPs).

### GERMLINE Analysis

IBD segments were detected using GERMLINE [18]. For this analysis, we phased the data from all relevant populations using Beagle [34] and then ran GERMLINE in genotype extension mode on a combined dataset of Roma, HapMap, India Project, POPRES and HGDP (*n* = 1,966 and 205,710 SNPs). We applied the following parameters for calculating IBD segments: seed size = 75, minimum IBD segments length = 3 cM, and the number of heterozygous or homozygous errors = 0. The output of GERMLINE was used to compute an average pairwise sharing between populations *I* and *J* as previously reported in reference [35].
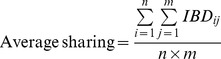
where 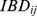 = the length of IBD segment shared between individual *i* and *j* and *n*, *m* are the number of individuals in population *I* and *J* respectively.

For identifying the source of the European ancestry, we computed the average sharing between Roma and each of the four geographic regions in Europe. Each group contained the following samples: *Northern-Europe* (*n* = 595) included CEU from HapMap, Orcadian from HGDP, and Latvia, United Kingdom, Ireland, Sweden, Scotland, Norway, Denmark, and Finland from POPRES, *Southern-Europe* (*n* = 649) included TSI from HapMap, Italian, Basque, Sardinian, and Tuscan from HGDP, and Spain, Croatia, Bosnia-Herzegovina, Albania, Macedonia, Slovenia, Kosovo, Italy, Cyprus, Portugal, Greece, and Serbia from POPRES, *Eastern-Europe* (*n* = 82) included Russian from HGDP and Romania, Hungary, Slovakia, Czech Republic, Bulgaria, Ukraine, Poland, and Russia from POPRES, and *Western-Europe* (*n* = 241) included French from HGDP and Germany, Belgium, France, Austria, and Netherlands from POPRES.

Similarly, for identifying the source of the South Asian ancestry we computed average IBD distance between Roma and South Asians. We grouped the South Asian samples in seven regional categories as follows: *North* (*n* = 38) included Tharu, Kharia, Vaish, Srivastava, Sahariya, Lodi, HGDP Pathan and Sindhi, *Northwest* (*n* = 225) included Kashmiri Pandit and POPRES Punjabi, *Northeast* (*n* = 8) included Nyshi and Ao Naga, *Southwest* (*n* = 16) included Kurumba and Hallaki, *Southeast* (*n* = 29) includes Madiga, Mala, Vysya, Chenchu, Naidu, Velama and Kamsali, *West* (*n* = 32) included Bhil, Meghawal and POPRES Gujarati, *East* included Santhal and Satnami, and *Andamanese* (*n* = 16) included Great Andamanese and Onge.

### Estimation of a Date of Mixture

We applied modified *ROLLOFF* [16] to estimate the date of mixture in a combined dataset containing 1,274 individuals and 524,053 SNPs. For each pair of SNPs (*x,y*) separated by a distance *d* Morgans, we compute covariance between (*x,y*). Specifically, we use the following statistic -
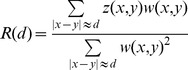
where 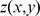 = covariance between SNPs (*x, y*) and weight function 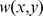 = a weight function that can be the allele frequency difference between the ancestral populations or the PCA based loadings for SNPs (*x, y*). We study the relationship of the weighted covariance with genetic distance, and obtain a date by fitting an exponential function with an affine term 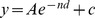, where *n* is the number of generations since admixture, *d* is the distance in Morgans, *c* is the affine term (non-zero asymptote of the fitted curve) and *A* is amplitude of the weighted LD curve (LD at short distances). Standard errors were computed using a weighted Block Jackknife [32], [33] where one chromosome was dropped in each run. We fit a sum of exponentials to estimate the dates of admixture under a two-pulse model of admixture using the exponential function: 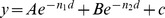, where *n_1_*, *n_2_* are the admixture dates in generations.

### Estimating Individual Autozygosity

We used PLINK v1.07 [20] to identify autozygous segments in the genome in a combined dataset of 1,274 individuals and 524,053 SNPs. PLINK uses a sliding window approach to find regions of the genome that are at least 1 MB in length and contains 100 contiguous homozygous SNPs. We allowed one heterozygous and five missing calls per segment. Autozygous segments were identified separately for each individual. We applied this method to compute genomic autozygosity (overall length of autozygous segments) for each Roma and 30 random individuals from each HapMap population.

### Estimating a Date for the Founder Event

To infer the date of the founder event, we compute the correlation of allele sharing as a measure of LD as described in reference [14] using a dataset containing Roma and HapMap populations (*n* = 1,142 and 726,404 SNPs). Specifically, we compute the autocorrelation of allele sharing between pairs Roma individuals, and then subtract the (Roma, CEU) cross-population autocorrelation to remove the effects of ancestral allele sharing. We thus get a measure for the Roma-specific LD related to the excess of allele sharing in this group. We plot the auto-correlation against genetic distance to infer the time of founder event. Specifically, we fit the exponential function:_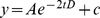,_ where *D* = distance in Morgans and *t* = time of founder event.

## Supporting Information

Figure S1 **ADMIXTURE Analysis.** To study the relationship of Roma with worldwide populations, we performed ADMIXTURE analysis. Each vertical line represents an individual colored based on the proportion of estimated ancestry for each cluster. (a) ADMIXTURE Analysis (K = 2 to K = 7) of Roma and HapMap populations. Lowest cross validation error was observed for K = 6; (b) ADMIXTURE Analysis of Roma, Europeans (CEU) and South Asians. Lowest cross validation error was observed for K = 3. We limit the sample size of all groups (except Roma) to 20 individuals.
(TIF)

Figure S2 **Estimating the proportion of West Eurasian and South Asian ancestry in Roma.** In order to estimate the proportion of West Eurasian ancestry in Roma, we use the phylogenetic tree shown below. The different colored lines show drift that has occurred between the populations connected by the line. The orange line shows the drift between YRI and Adygei and the red and green lines shows the drift separating Roma and Onge. *m* denotes the shared drift between Roma and Onge. See methods for details for estimating the West Eurasian ancestry proportion (*p*) in Roma that derives from India (ANI) and Europe (post exodus from India). This figure is adapted from Reich et al (2009).
(TIF)

Figure S3
**Normalization term from original** ***ROLLOFF*** **correlation coefficient formulation.** We plot the squared normalization term 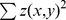
as a function of genetic distance *d* between SNPs for the admixture plus bottleneck scenarios described in Table S3, using either the correlation (a) or covariance (b) versions of 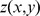. In the case of no bottleneck, the normalization term is dominated by finite sampling noise and exhibits no dependence on *d*. For the cases of a strong bottleneck post-admixture, however, 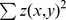 exhibits an exponential decay 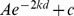 with rate constant approximately equal to twice the age of the bottleneck ((a) best-fit *k* = 15, 25, 46, 65, 83 and (b) *k* = 12, 20, 41, 60, 78 shown as solid lines).(TIF)

Figure S4 ***ROLLOFF*** **Simulation Results: Variable age of mixture.** We simulated data for 25 admixed individuals with mixed European and East Asian ancestry where the proportion of European ancestry was set to 20% and the admixture date was set between 10–300 generations (as shown below). We ran the *ROLLOFF* (using *R(d)*) to estimate the date of mixture using allele frequencies in an independent dataset of French and East Asians. Standard errors were computed using weighted block jackknife as described in the Methods.
(TIF)

Figure S5 ***ROLLOFF*** **Simulation using PCA-loadings.** We simulated data for admixed individuals with mixed European and East Asian ancestry where the proportion of European ancestry was set to 80% (similar to Roma) and the mixture occurred 30 generations ago (left panel: *n* = 27) and 100 generations ago (right panel: *n* = 27). We ran *ROLLOFF* (using *R(d)*) to estimate the date of mixture in this panel of individuals using the PCA-based loadings computed using an CEU and an independent dataset containing simulated data for 3 admixed groups with European ancestry equal to 30%, 50% and 70%. We estimated that the dates of mixture were 33±1 generation for the left panel (true date = 30 generations), and 99±4 generations for right panel (true date = 100 generations).
(TIF)

Figure S6 **IBD Sharing of Roma with European populations.** We computed average pairwise IBD sharing between Roma from European samples (from POPRES, HapMap and HGDP datasets) clustered based on geography.
(TIF)

Figure S7 **Bootstrap analysis to compute error in IBD statistics.** We performed bootstrap analysis where we randomly sample up to 30 individuals from each of the 8 South Asian regional groups and compute average pairwise IBD between Roma and South Asians. We performed a total of 100 runs and obtained the mean and standard error for the IBD statistic (vertical bars shown). For regional groups which had less than 30 samples (such as Northeast, Southwest, East, and Andamanese), all samples were included in each run and so no standard errors are shown.
(TIF)

Table S1 **Average frequency differentiation (F_st_) for Roma and HapMap populations.**
(DOC)

Table S2 **Formal tests of admixture.**
(DOC)

Table S3 **Simulations for estimating dates of admixture events: Founder events post admixture model.**
(DOC)

Table S4 **Simulations for estimating dates of admixture events: Model with two gene flow events.**
(DOC)

Table S5 **Simulations for estimating dates of founder events.**
(DOC)

Note S1 **New** ***ROLLOFF*** **Statistic.**
(PDF)

Note S2 **Simulations for estimating dates of admixture events.**
(PDF)

Note S3 **Computing corrected IBD sharing distance between Roma and South Asian groups.**
(PDF)

Note S4 **Simulations for estimating date of founder event.**
(PDF)
