# Supplementary figures and images for: Reconstructing Roma History from Genome-Wide Data
Source: PLoS One. 2013 Mar 13;8(3):e58633. doi: 10.1371/journal.pone.0058633 (PMC3596272; doi:10.1371/journal.pone.0058633)

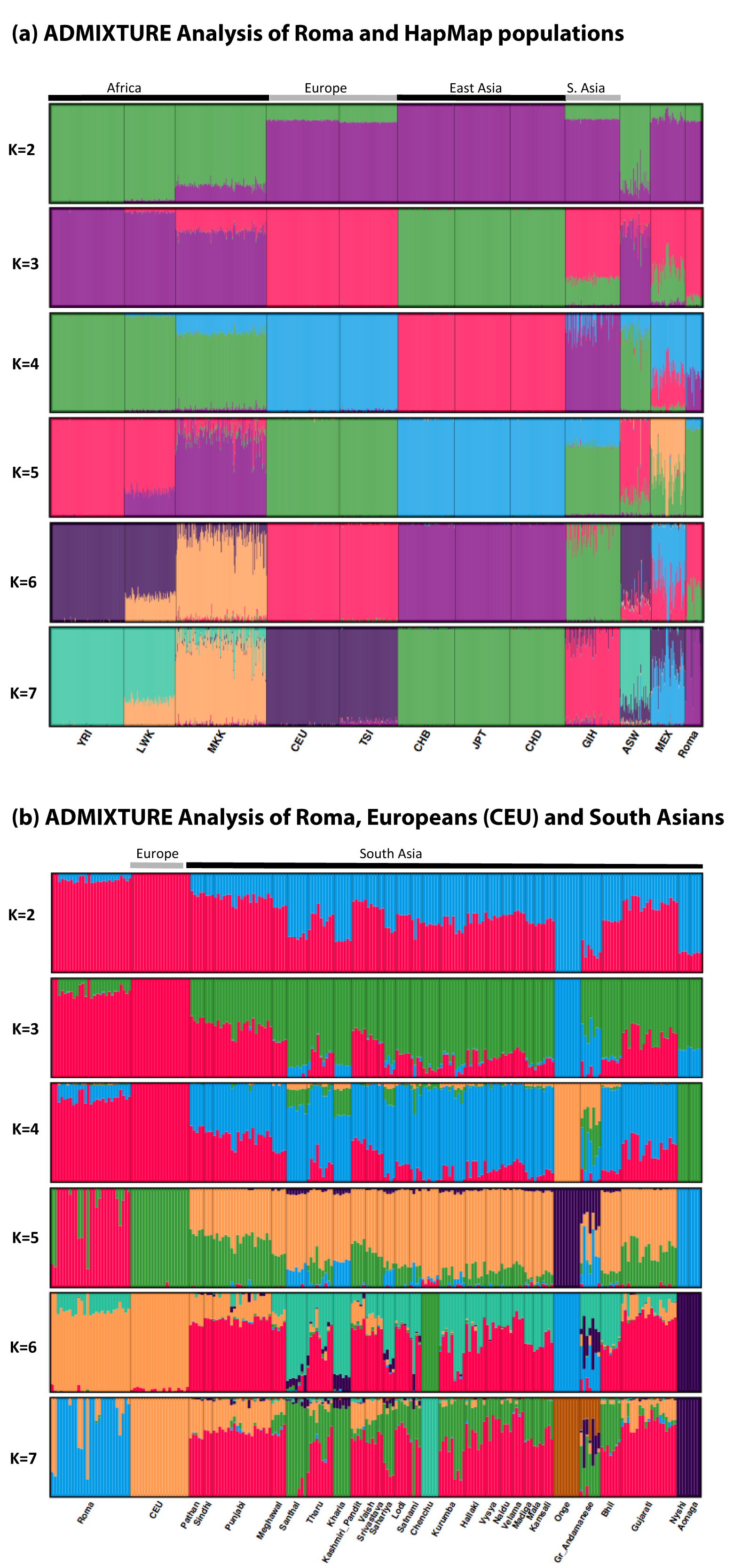

Supplement: Figure S1 — ADMIXTURE Analysis. To study the relationship of Roma with worldwide populations, we performed ADMIXTURE analysis. Each vertical line represents an individual colored based on the proportion of estimated ancestry for each cluster. (a) ADMIXTURE Analysis (K = 2 to K = 7) of Roma and HapMap populations. Lowest cross validation error was observed for K = 6; (b) ADMIXTURE Analysis of Roma, Europeans (CEU) and South Asians. Lowest cross validation error was observed for K = 3. We limit the sample size of all groups (except Roma) to 20 individuals. (TIF) [file pone.0058633.s001.tif]

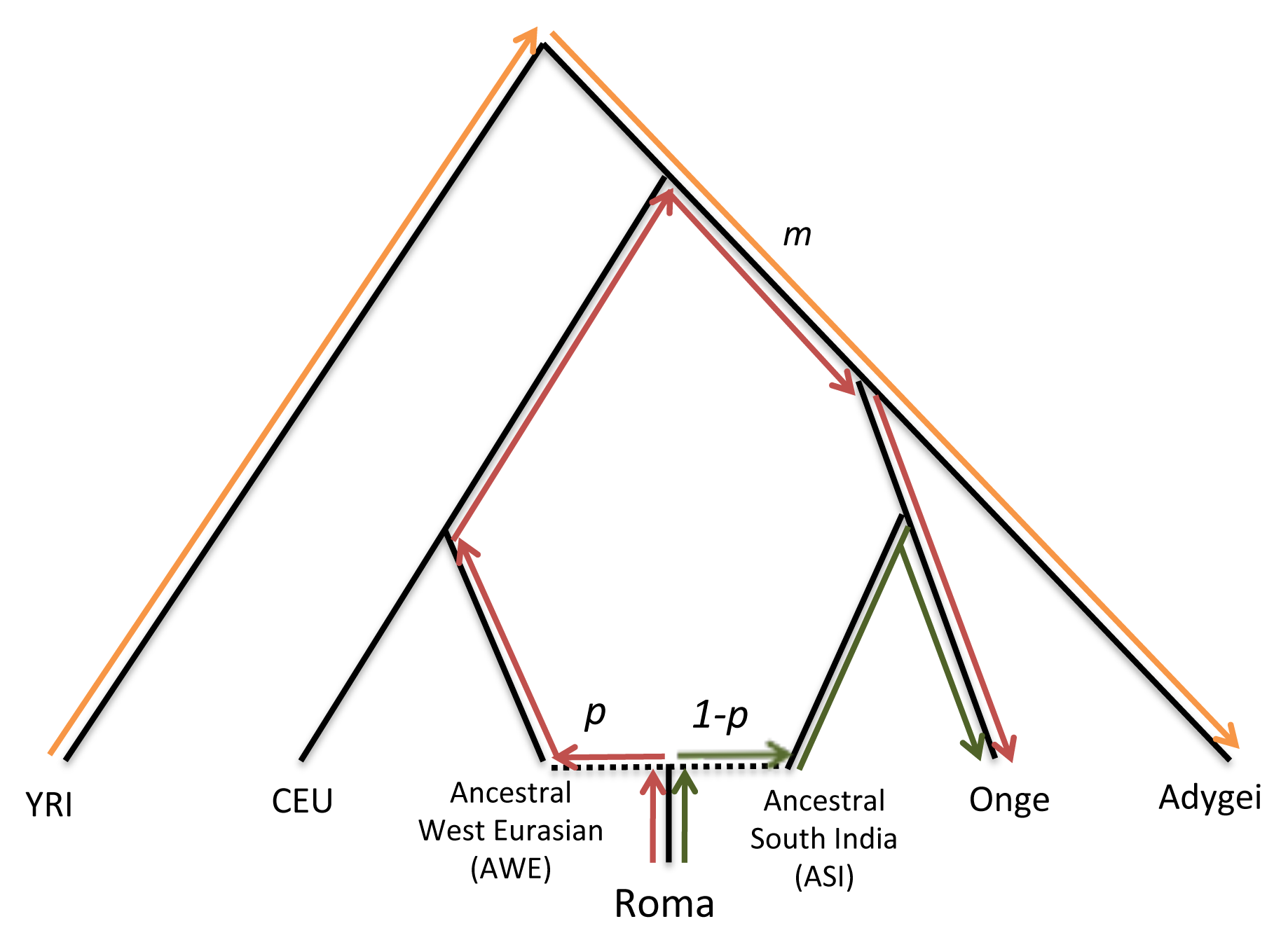

Supplement: Figure S2 — Estimating the proportion of West Eurasian and South Asian ancestry in Roma. In order to estimate the proportion of West Eurasian ancestry in Roma, we use the phylogenetic tree shown below. The different colored lines show drift that has occurred between the populations connected by the line. The orange line shows the drift between YRI and Adygei and the red and green lines shows the drift separating Roma and Onge. m denotes the shared drift between Roma and Onge. See methods for details for estimating the West Eurasian ancestry proportion (p) in Roma that derives from India (ANI) and Europe (post exodus from India). This figure is adapted from Reich et al (2009). (TIF) [file pone.0058633.s002.tif]

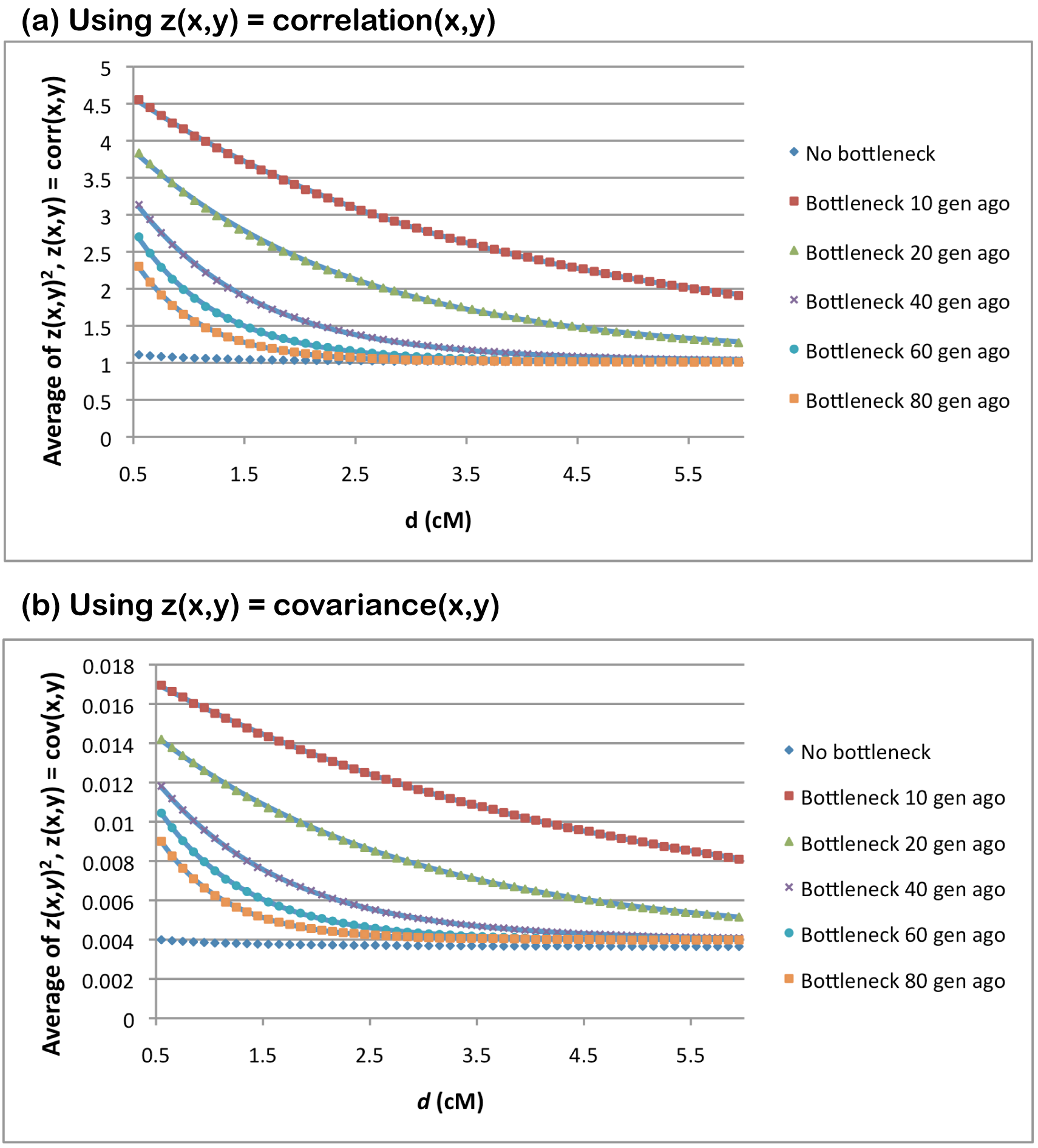

Supplement: Figure S3 — Normalization term from original ROLLOFF correlation coefficient formulation. We plot the squared normalization term as a function of genetic distance d between SNPs for the admixture plus bottleneck scenarios described in Table S3, using either the correlation (a) or covariance (b) versions of . In the case of no bottleneck, the normalization term is dominated by finite sampling noise and exhibits no dependence on d. For the cases of a strong bottleneck post-admixture, however, exhibits an exponential decay with rate constant approximately equal to twice the age of the bottleneck ((a) best-fit k = 15, 25, 46, 65, 83 and (b) k = 12, 20, 41, 60, 78 shown as solid lines). (TIF) [file pone.0058633.s003.tif]

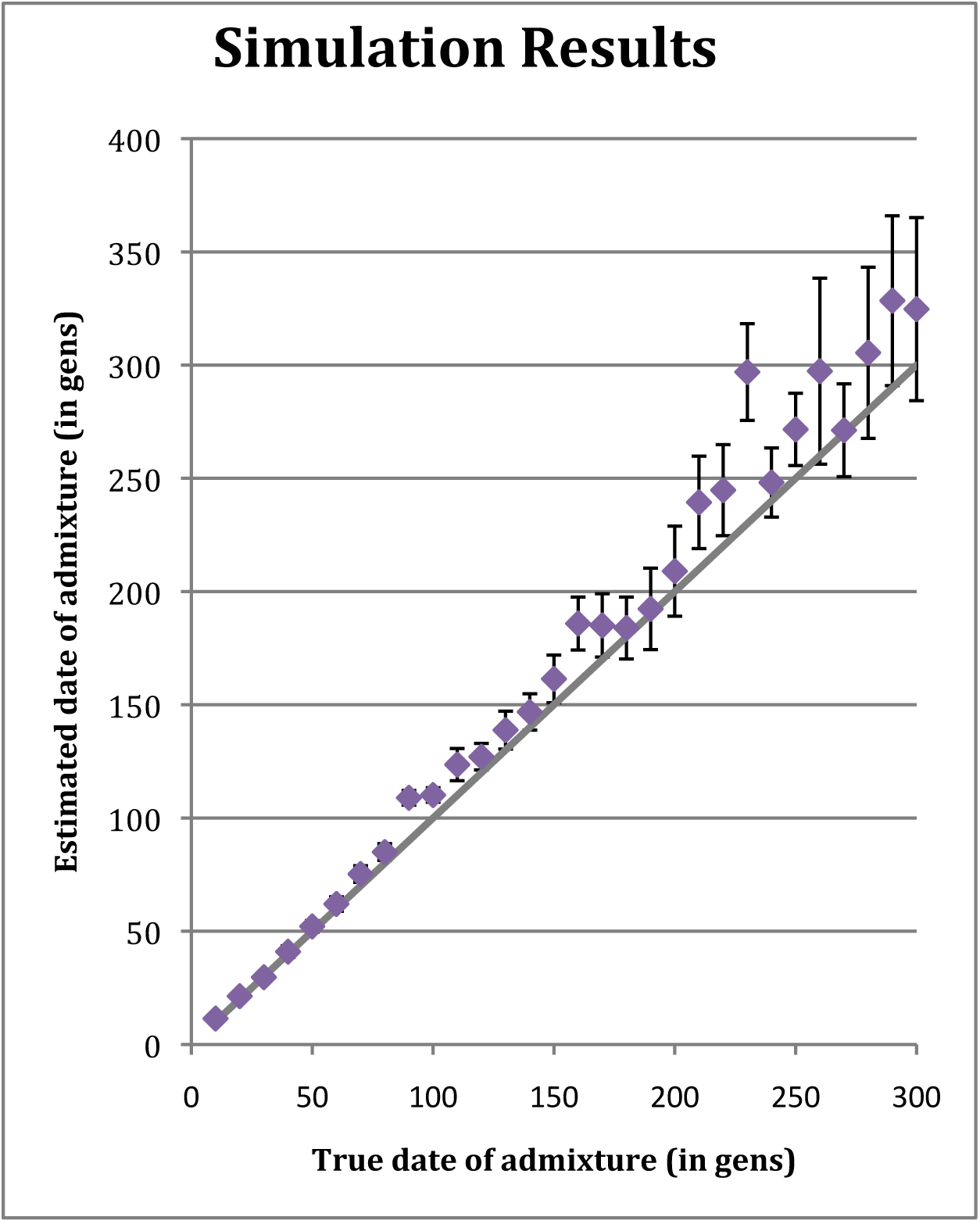

Supplement: Figure S4 — ROLLOFF Simulation Results: Variable age of mixture. We simulated data for 25 admixed individuals with mixed European and East Asian ancestry where the proportion of European ancestry was set to 20% and the admixture date was set between 10–300 generations (as shown below). We ran the ROLLOFF (using R(d)) to estimate the date of mixture using allele frequencies in an independent dataset of French and East Asians. Standard errors were computed using weighted block jackknife as described in the Methods. (TIF) [file pone.0058633.s004.tif]

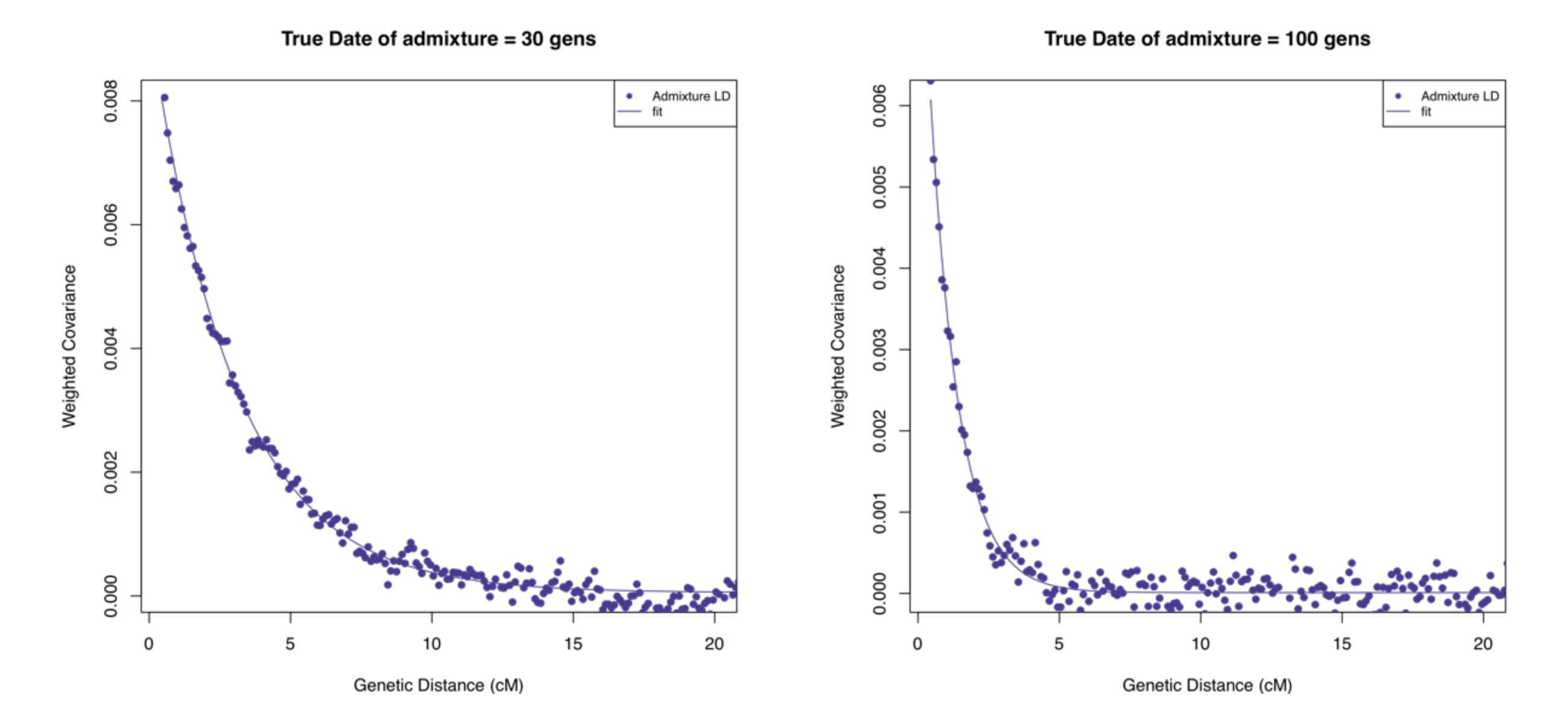

Supplement: Figure S5 — ROLLOFF Simulation using PCA-loadings. We simulated data for admixed individuals with mixed European and East Asian ancestry where the proportion of European ancestry was set to 80% (similar to Roma) and the mixture occurred 30 generations ago (left panel: n = 27) and 100 generations ago (right panel: n = 27). We ran ROLLOFF (using R(d)) to estimate the date of mixture in this panel of individuals using the PCA-based loadings computed using an CEU and an independent dataset containing simulated data for 3 admixed groups with European ancestry equal to 30%, 50% and 70%. We estimated that the dates of mixture were 33±1 generation for the left panel (true date = 30 generations), and 99±4 generations for right panel (true date = 100 generations). (TIF) [file pone.0058633.s005.tif]

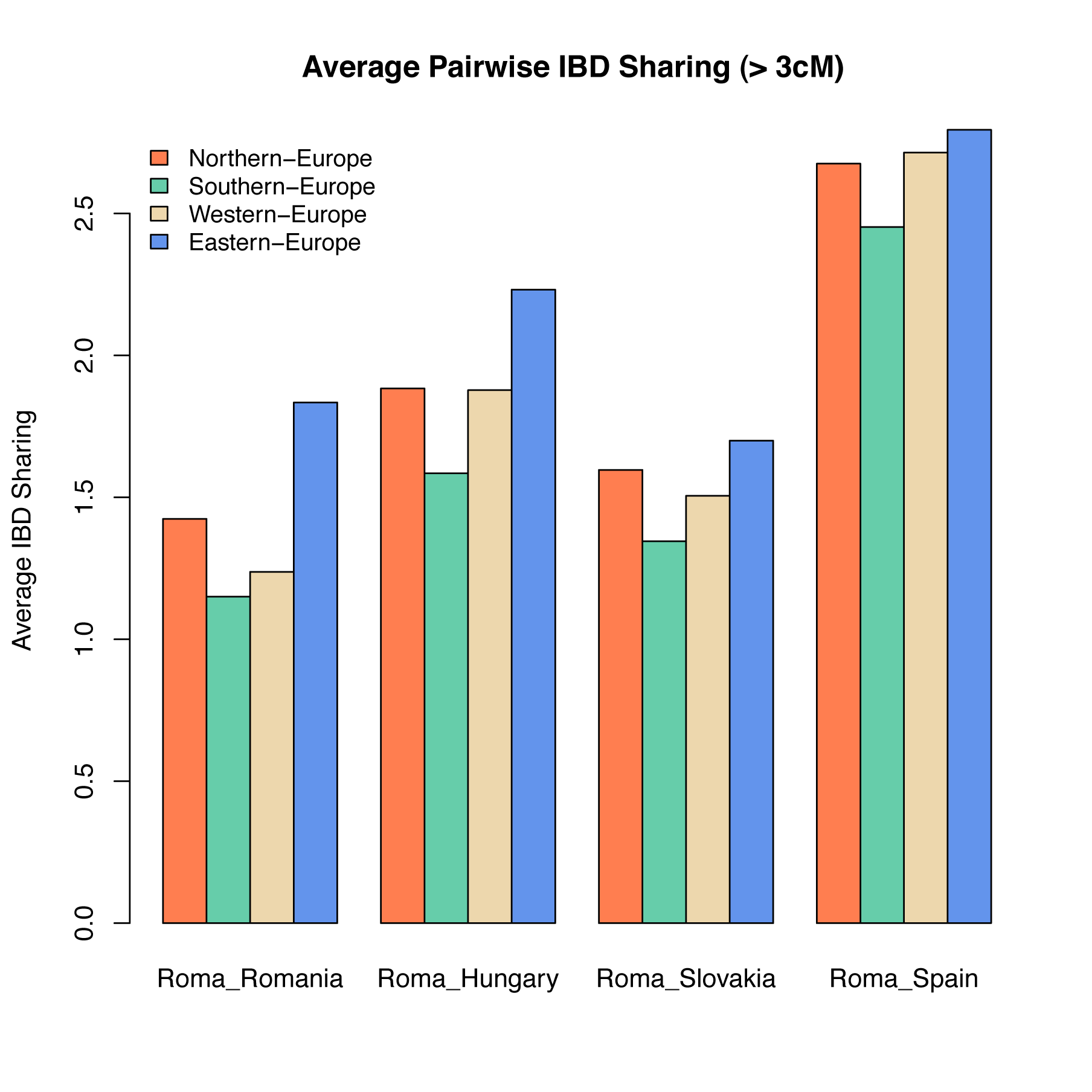

Supplement: Figure S6 — IBD Sharing of Roma with European populations. We computed average pairwise IBD sharing between Roma from European samples (from POPRES, HapMap and HGDP datasets) clustered based on geography. (TIF) [file pone.0058633.s006.tif]

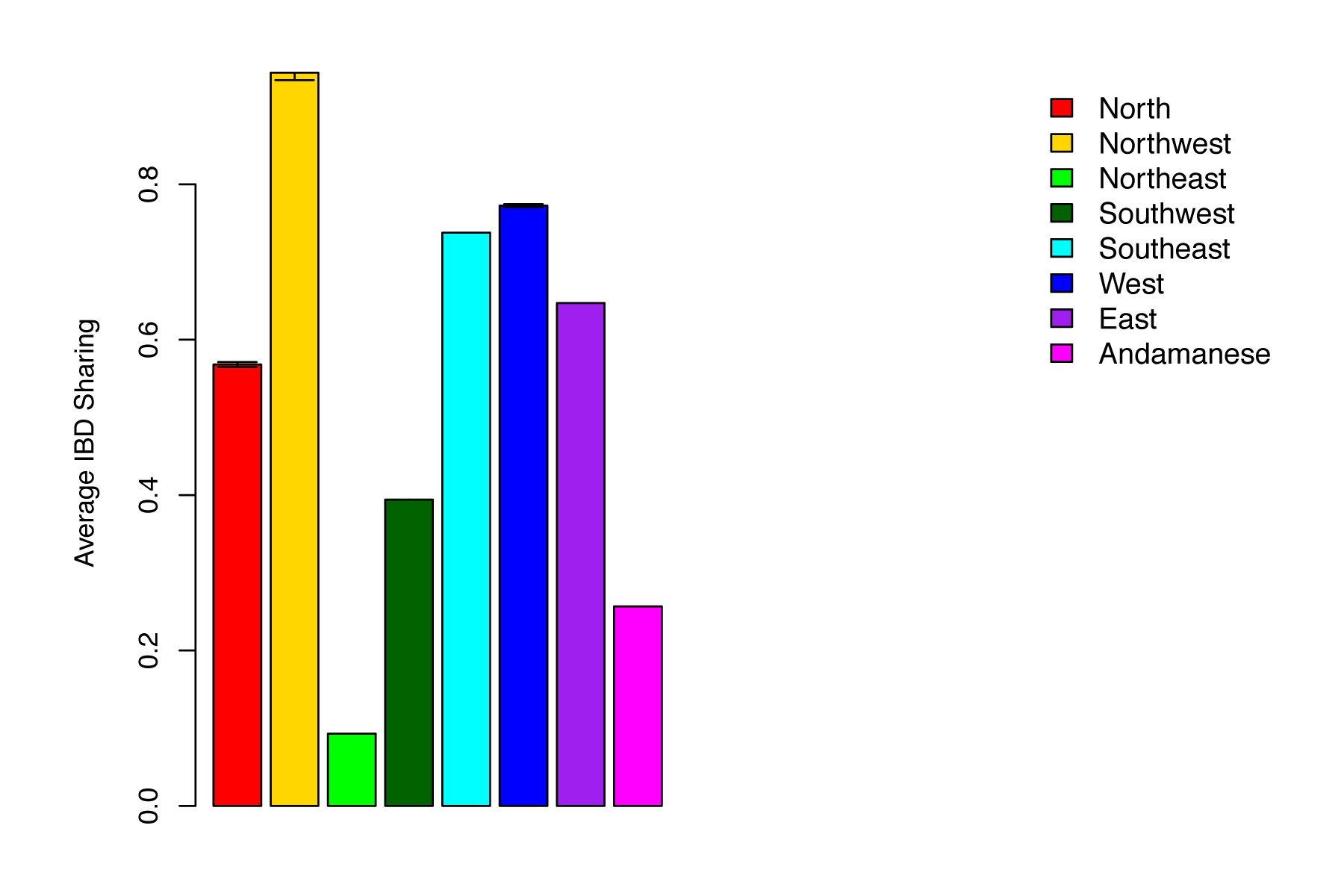

Supplement: Figure S7 — Bootstrap analysis to compute error in IBD statistics. We performed bootstrap analysis where we randomly sample up to 30 individuals from each of the 8 South Asian regional groups and compute average pairwise IBD between Roma and South Asians. We performed a total of 100 runs and obtained the mean and standard error for the IBD statistic (vertical bars shown). For regional groups which had less than 30 samples (such as Northeast, Southwest, East, and Andamanese), all samples were included in each run and so no standard errors are shown. (TIF) [file pone.0058633.s007.tif]
