# Supplementary material for: Reconstructing Roma History from Genome-Wide Data
Source: PLoS One. 2013 Mar 13;8(3):e58633. doi: 10.1371/journal.pone.0058633 (PMC3596272; doi:10.1371/journal.pone.0058633)
Supplement: Table S1 — Average frequency differentiation (Fst) for Roma and HapMap populations. (DOC) [file pone.0058633.s008.doc]

**Table S1. Average frequency differentiation (F_st_) for Roma and HapMap populations.**

|  | **CEU** | **YRI** | **CHB** | **JPT** | **ASW** | **CHD** | **GIH** | **LWK** | **MEX** | **MKK** | **TSI** | **Roma** |
| --- | --- | --- | --- | --- | --- | --- | --- | --- | --- | --- | --- | --- |
| **CEU** | 0 | 0.14 | 0.102 | 0.104 | 0.088 | 0.103 | 0.033 | 0.13 | 0.036 | 0.093 | 0.003 | 0.016 |
| **YRI** | 0.14 | 0 | 0.169 | 0.17 | 0.008 | 0.169 | 0.129 | 0.007 | 0.134 | 0.025 | 0.136 | 0.135 |
| **CHB** | 0.102 | 0.169 | 0 | 0.007 | 0.127 | 0.001 | 0.071 | 0.159 | 0.064 | 0.131 | 0.102 | 0.092 |
| **JPT** | 0.104 | 0.17 | 0.007 | 0 | 0.129 | 0.008 | 0.072 | 0.161 | 0.065 | 0.133 | 0.104 | 0.094 |
| **ASW** | 0.088 | 0.008 | 0.127 | 0.129 | 0 | 0.128 | 0.083 | 0.009 | 0.088 | 0.013 | 0.086 | 0.087 |
| **CHD** | 0.103 | 0.169 | 0.001 | 0.008 | 0.128 | 0 | 0.071 | 0.16 | 0.066 | 0.132 | 0.103 | 0.093 |
| **GIH** | 0.033 | 0.129 | 0.071 | 0.072 | 0.083 | 0.071 | 0 | 0.119 | 0.038 | 0.086 | 0.032 | 0.026 |
| **LWK** | 0.13 | 0.007 | 0.159 | 0.161 | 0.009 | 0.16 | 0.119 | 0 | 0.125 | 0.015 | 0.126 | 0.125 |
| **MEX** | 0.036 | 0.134 | 0.064 | 0.065 | 0.088 | 0.066 | 0.038 | 0.125 | 0 | 0.093 | 0.037 | 0.04 |
| **MKK** | 0.093 | 0.025 | 0.131 | 0.133 | 0.013 | 0.132 | 0.086 | 0.015 | 0.093 | 0 | 0.088 | 0.089 |
| **TSI** | 0.003 | 0.136 | 0.102 | 0.104 | 0.086 | 0.103 | 0.032 | 0.126 | 0.037 | 0.088 | 0 | 0.015 |
| **Roma** | 0.016 | 0.135 | 0.092 | 0.094 | 0.087 | 0.093 | 0.026 | 0.125 | 0.04 | 0.089 | 0.015 | 0 |
