# Supplementary material for: Reconstructing Roma History from Genome-Wide Data
Source: PLoS One. 2013 Mar 13;8(3):e58633. doi: 10.1371/journal.pone.0058633 (PMC3596272; doi:10.1371/journal.pone.0058633)
Supplement: Table S2 — Formal tests of admixture. (DOC) [file pone.0058633.s009.doc]

**Table S2. Formal tests of admixture.**

| **Population (*X*)** | **Sam-ples** | **Region** | ***Z*-score for *4 Population test*** | | | **Estimated West Eurasian Ancestry %** |
| --- | --- | --- | --- | --- | --- | --- |
|  |  |  | **(P_CEU_-P_YRI_) × (P_Onge_- P_X_)** | **(P_YRI_-P_Onge_)**  **× (P_CEU_-P_X_)** | **(P_x_-P_YRI_)** |  |
|  |  |  |  |  | **× (P_CEU_-P_Onge_)** |  |
| Roma | 18 | Hungary | -33 | 4.8 | -29.3 | 78.3 ± 1.9% |
| Roma* | 3 | Slovakia | -26.6 | 3.5 | -22.8 | 71.5 ± 3.1% |
| Roma** | 1 | Romania | -20.2 | 0.7 | -19.2 | 79.4 ± 4.7% |
| Roma | 2 | Spain | -25.3 | 0.9 | -24 | 75.6 ± 4.0% |
| Roma | 24 | Combined | -33 | 4.8 | -29.5 | 77.5 ± 1.8% |

*NOTE:* * indicates that some samples from the group appear to have recent European gene flow. These samples were excluded from the analysis (the number of * indicates the number of samples excluded). Ancestry proportions were estimates based on *f_4_ Ratio Estimation* using Yoruba, Adygei, Europeans (CEU) and Onge as the reference populations.
