# Supplementary material for: Reconstructing Roma History from Genome-Wide Data
Source: PLoS One. 2013 Mar 13;8(3):e58633. doi: 10.1371/journal.pone.0058633 (PMC3596272; doi:10.1371/journal.pone.0058633)
Supplement: Table S3 — Simulations for estimating dates of admixture events: Founder events post admixture model. (DOC) [file pone.0058633.s010.doc]

**Table S3. Simulations for estimating dates of admixture events: Founder events post admixture model.**

| **True date of admixture** | **True date of founder event (*x*)** | **Date based on original *ROLLOFF* statistic (a)** | **Date based on modified *ROLLOFF* statistic (b)** | **Date based on modified *ROLLOFF* statistic (c)** |
| --- | --- | --- | --- | --- |
| 30 | N/A | 31.3 | 32.0 | 32.1 |
| 30 | 5 | 24.6 | 30.1 | 29.0 |
| 30 | 10 | 27.7 | 34.1 | 32.3 |
| 30 | 20 | 23.3 | 32.7 | 31.0 |
| 30 | 25 | 23.4 | 30.8 | 29.5 |
|  |  |  |  |  |
| 100 | N/A | 94.1 | 96.8 | 97.0 |
| 100 | 10 | 93.9 | 106.1 | 102.9 |
| 100 | 20 | 87.1 | 102.7 | 97.3 |
| 100 | 40 | 75.3 | 95.6 | 92.2 |
| 100 | 60 | 83.9 | 106.3 | 102.8 |
| 100 | 100 | 81.6 | 101.1 | 99.0 |

Note: We simulated data from three populations Pop A (n = 20), Pop B (n = 20) and Pop C (n = 30) using MaCS coalescent simulator. Populations A and B diverged 1800 generations ago. The effective population size for all populations was set 12,500 at all times (except during the founder event). The mutation and recombination rates were set to 2x10^-8^ and 1x10^-8^ per base pair per generation. Pop C can be considered as an admixed population that has ancestry 60%/40% ancestry from A’ and B’ (admixture time (t) is set to 30/ 100 generations). Pop A’ and A diverged 120 generations and B’ and B diverged 200 generations ago. At generation *x* (shown in table above), Pop C undergoes a severe founder event where the effective population size reduces to 5 individuals for one generation. When *x* = N/A, there was no founder event. We performed *ROLLOFF* (using original and modified statistic) with Pop C as the target and Pop A and B as the reference populations. We performed 5 replicates for each parameter and report the average estimated date of mixture. The statistics used were -

(a) Original *ROLLOFF* Statistic: ; where *z*(*x*,*y*) = correlation between *x* and *y*.

(b) Modified Statistic: ; where *z*(*x*,*y*) = correlation between *x* and *y*.

(c) Modified Statistic: ;where *z*(*x*,*y*) = ***covariance*** between *x* and *y*.
