# Supplementary material for: Reconstructing Roma History from Genome-Wide Data
Source: PLoS One. 2013 Mar 13;8(3):e58633. doi: 10.1371/journal.pone.0058633 (PMC3596272; doi:10.1371/journal.pone.0058633)
Supplement: Table S4 — Simulations for estimating dates of admixture events: Model with two gene flow events. (DOC) [file pone.0058633.s011.doc]

**Table S4. Simulations for estimating dates of admixture events: Model with two gene flow events**.

| **Date of first wave of mixture (λ_1_)** | **Date of second wave of mixture (λ_2_)** | **Estimated date in generations (± standard error)** |
| --- | --- | --- |
| 120 | 20 | 36 ± 3 |
| 170 | 20 | 28 ± 2 |
| 220 | 20 | 23 ± 2 |
| 270 | 20 | 24 ± 2 |
| 320 | 20 | 25 ± 1 |
| 370 | 20 | 25 ± 1 |
| 420 | 20 | 22 ± 1 |
|  |  |  |
| 130 | 30 | 46 ± 3 |
| 180 | 30 | 47 ± 3 |
| 230 | 30 | 41 ± 2 |
| 280 | 30 | 39 ± 2 |
| 330 | 30 | 39 ± 3 |
| 380 | 30 | 35 ± 2 |
| 430 | 30 | 32 ± 3 |

Note: We simulated 27 individuals using CEU and Han Chinese as the ancestral populations where we set the overall European ancestry proportion to be 80%. We then performed *ROLLOFF* (using *R(d))* with an independent dataset of Europeans (HGDP French) and East Asians (HapMap CHB) as reference populations.
