# Supplementary material for: Reconstructing Roma History from Genome-Wide Data
Source: PLoS One. 2013 Mar 13;8(3):e58633. doi: 10.1371/journal.pone.0058633 (PMC3596272; doi:10.1371/journal.pone.0058633)
Supplement: Table S5 — Simulations for estimating dates of founder events. (DOC) [file pone.0058633.s012.doc]

**Table S5. Simulations for estimating dates of founder events.**

| **Simulation scenario** | **True date of founder event** | **True date of admixture** | **Estimated date of founder event**  **(in generations)** |
| --- | --- | --- | --- |
| **Founder event only** | | | |
|  | 10 | -- | 11.2 |
|  | 20 | -- | 20.8 |
|  | 40 | -- | 39.3 |
|  | 60 | -- | 52.7 |
|  | 80 | -- | 74.9 |
|  | 100 | -- | 95.7 |
|  |  |  |  |
| **Founder event + Admixture** | | | |
|  | 10 | 10 | 8.2 |
|  | 10 | 20 | 8.4 |
|  | 10 | 40 | 8.3 |
|  | 10 | 60 | 9.2 |
|  | 10 | 80 | 11.8 |
|  | 10 | 100 | 9.9 |
|  | 30 | 10 | 24.4 |
|  | 30 | 20 | 29.9 |
|  | 30 | 30 | 30.1 |
|  | 30 | 40 | 26.5 |
|  | 30 | 60 | 26.2 |
|  | 30 | 80 | 27.9 |
|  | 30 | 100 | 27.6 |
|  | 100 | 10 | 50 |
|  | 100 | 20 | 60.9 |
|  | 100 | 40 | 67.4 |
|  | 100 | 60 | 81.5 |
|  | 100 | 80 | 113.3 |
|  | 100 | 100 | 92.7 |
|  | 100 | 150 | 85.3 |

Note: We simulated 20 individuals from Pop A and 25 individuals from Pop B using MaCS coalescent simulator. The two populations diverged 1800 generations ago. The effective population size for both populations was set 12,500 at all times (except during the founder event). The mutation and recombination rates were set to 2x10^-8^ and 1x10^-8^ per base pair per generation. During the founder event, the effective population size reduced to 5 individuals for one generation at the date specified in the table above. For each simulation we generated data for ~450,000 polymorphic sites. SNPs with minor allele frequencies of <1% were discarded.
