# Supplementary material for: Reconstructing Roma History from Genome-Wide Data
Source: PLoS One. 2013 Mar 13;8(3):e58633. doi: 10.1371/journal.pone.0058633 (PMC3596272; doi:10.1371/journal.pone.0058633)
Supplement: Note S1 — New ROLLOFF Statistic. (PDF) [file pone.0058633.s013.pdf]

NOTE S1. New *ROLLOFF* statistic

In this note, we consider alternative forms of the *ROLLOFF* linkage disequilbrium (LD) statistic [1] for dating population admixture events. We show that the original *ROLLOFF* statistic is susceptible to downward bias in the event of a recent population bottleneck, and we propose a modification of the statistic that is robust against such an effect (Table S3).

The *ROLLOFF* technique applies two key insights: first, that admixture creates LD that decays exponentially as recombination occurs—explicitly, as e^{-nd}, where n is the number of generations since admixture and d is the genetic distance between SNPs—and second, that the amount of admixture LD between each pair of SNPs is proportional to the product of the allele frequency divergences between the ancestral populations at those sites. The latter observation allows the e^{-nd} admixture LD decay signal to be detected (via a SNP-pair weighting scheme) and harnessed to infer the mixture date n.

The original *ROLLOFF* statistic captures admixture LD in the form of SNP autocorrelation. Defining $z(x,y)$ to be the (Fisher z-transformed) correlation coefficient between SNP genotypes at sites x and y, *ROLLOFF* computes the correlation coefficient between values of $z(x,y)$ and weights $w(x,y)$ over pairs of SNPs binned by genetic distance:

$$A(d) := \frac{\sum_{|x-y|\approx d} z(x,y)w(x,y)}{\sqrt{\sum_{|x-y|\approx d} z(x,y)^2}\sqrt{\sum_{|x-y|\approx d} w(x,y)^2}}, \tag{1}$$

the idea being that $A(d) \propto e^{-nd}$.

While this setup estimates accurate dates for typical admixture scenarios, it turns out to be noticeably biased in the case of a recent bottleneck. However, we will show that the

following modified statistic does not suffer from the bias:

$$R(d) := \frac{\sum_{|x-y|\approx d} z(x,y)w(x,y)}{\sum_{|x-y|\approx d} w(x,y)^2}. \quad (2)$$

(Note that $R(d)$ amounts to taking the regression coefficient of $z(x,y)$ against the weights $w(x,y)$ for SNP pairs within each bin).

An additional detail of our modified *ROLLOFF* statistic is that we change $z(x,y)$ to measure admixture LD as the covariance between SNPs x and y rather than the correlation (i.e., it equals the classical LD statistic D rather than the correlation r). We believe the use of covariance rather than correlation for $z(x,y)$ has little impact on the performance and properties of the statistic (as it roughly amounts to multiplying by a constant factor) but makes the statistic more amenable to mathematical analysis.

Explanation of bias from recent bottlenecks

The bias in the original formulation of *ROLLOFF* (1) introduced by a recent bottleneck can be readily explained at an intuitive level: the problem is that while the numerator of the correlation coefficient, $\sum_{|x-y|\approx d} z(x,y)w(x,y)$, decays as e^{-nd} as intended, the normalization term

$$\sqrt{\sum_{|x-y|\approx d} z(x,y)^2} \quad (3)$$

also exhibits a decay behavior that confounds the e^{-nd} signal (Figure S3). The reason is that a strong bottleneck introduces a very large amount of LD, effectively giving $z(x,y)$ a random large magnitude immediately post-bottleneck that is independent of the distance between x and y. This LD subsequently decays as e^{-nd} until the magnitude of $z(x,y)$ reaches the level of random sampling noise (arising from the finite sample of admixed individuals being used to calculate z). In non-bottlenecked cases, the square-norm of

$z(x,y)$ is usually dominated by sampling noise, so the normalization term (3) effectively amounts to a constant, and dividing by (3) has no effect on the decay rate of $A(d)$.

The "regression coefficient" version of the *ROLLOFF* statistic (2) does not contain the normalization term (3) and thus does not incur bias from bottlenecks.

Precise effect of genetic drift on the *ROLLOFF* statistics

We now rigorously derive the above intuition. We will assume in the following calculations that the *ROLLOFF* weights are taken as the product of allele frequency divergences $\delta(x)$ and $\delta(y)$ in the ancestral mixing populations:

$$w(x,y) := \delta(x)\delta(y).$$

Our reasoning below applies whether we have the true values of $\delta(x)$ and $\delta(y)$ or computed weights based on related reference populations or PCA loadings. We also assume that all SNPs are polymorphic ancestrally—i.e., we ignore mutations that have arisen in the admixed population—and that the SNP ascertainment is unbiased with respect to the populations under consideration.

For a diploid population of size N with chromosomes indexed by $i = 1, \ldots, 2N$, we set

$$z(x,y) := \frac{1}{2N}\sum_{i=1}^{2N}(X_i - \mu_x)(Y_i - \mu_y)$$

to be the covariance between binary alleles X_i and Y_i at sites x and y, respectively. We assume for ease of discussion that the data are phased; for unphased data, $z(x,y)$ is essentially a noisier version of the above because of cross terms.

We are primarily interested in the behavior of $z(x,y)$ from one generation to the next. Fix

a pair of SNPs x and y at distance d and let z_0 denote the value of $z(x,y)$ at a certain point in time. After one generation, due to finite population size and recombination, the covariance becomes [2]

$$z_1 = z_0 e^{-d}(1 - 1/2N) + \varepsilon, \tag{4}$$

where N is the population size, e^{-d} is the probability of no recombination, $(1 - 1/2N)$ is a Bessel correction, and ε is a noise term with mean 0 and variance on the order of $1/N$.

Iterating this equation over n generations, the final covariance is

$$z_n = z_0 e^{-nd} e^{-n/2N_e} + \varepsilon_{\text{agg}},$$

where N_e is the effective population size over the interval and ε_{agg} is a sum of n partially decayed noise terms.

Now let time 0 denote the time of admixture between two ancestral populations mixing in proportions α and $\beta := 1 - \alpha$. (The bottleneck may have occurred either before or after this point, as long as it does not influence the calculation of the weights). Then $E[z_0]$ is:

$$E[z_0] = 2\alpha\beta\delta(x)\delta(y),$$

Assuming the mixture is homogeneous and the distance d is large enough that background LD can be ignored. (In practice, heterogeneity in the admixed population changes the above form and results in the addition of an affine term to the *ROLLOFF* curve which we explicitly fit. We also typically fit only data from SNP pairs at distance $d > 0.5cM$ to avoid background LD) .

We can now compute the modified *ROLLOFF* statistic:

$$
\begin{aligned}
E[R(d)] &= E\left[\frac{\sum_{|x-y|\approx d} z(x,y)\delta(x)\delta(y)}{\sum_{|x-y|\approx d}\delta(x)^2\delta(y)^2}\right] \\
&\approx \frac{\sum_{|x-y|\approx d}[2\alpha\beta\delta(x)\delta(y)e^{-nd}e^{-n/2N_e}+\varepsilon_{\text{agg}}]\delta(x)\delta(y)}{\sum_{|x-y|\approx d}\delta(x)^2\delta(y)^2} \\
&\approx 2\alpha\beta e^{-nd}e^{-n/2N_e}.
\end{aligned}
$$

Importantly, in the last step we use the fact that the combined noise term ε_{agg} is uncorrelated with $\delta(x)\delta(y)$. Thus, even a strong bottleneck with a low value of N_e only scales $R(d)$ by the constant factor $e^{-n/2N_e}$, and the e^{-nd} scaling of the *ROLLOFF* curve as a function of d is unaffected.

On the other hand, if we use the original correlation form (1) of the *ROLLOFF* statistic $A(d)$, then the numerator still has the form of an exponential decay Ae^{-nd}, but now we divide this by the norm $\sqrt{\sum_{|x-y|\approx d} z(x,y)^2}$. In the case of a strong bottleneck, $z(x,y) = z_0e^{-nd}e^{-n/2N_e}+\varepsilon_{\text{agg}}$ can be dominated by the aggregate noise term ε_{agg}. Indeed, if the bottleneck occurred k generations ago, then the noise terms ε_i from the time of reduced population size will have decayed by e^{-kd} since the bottleneck but can still have large variance if the population size N_{bot} was very small at the time. In this case, at lower values of d, $E[z(x,y)^2] = E[(z_0e^{-nd}e^{-n/2N_e}+\varepsilon_{\text{agg}})^2]$ will be dominated by $E[\varepsilon_{\text{agg}}^2]$ which will scale approximately as e^{-2kd}/N_{bot}. Hence, the denominator of $A(d)$ will be significantly larger at low d than at high d, causing a partial cancellation of the exponential decay of the *ROLLOFF* curve and thus a downward bias in the estimated date of admixture.

References

1. Moorjani P, Patterson N, Hirschhorn JN, Keinan A, Hao L, et al. (2011) The History of African Gene Flow into Southern Europeans, Levantines, and Jews. PLoS Genetics 7: e1001373.

2. Hill, W, Robertson A. (1968) Linkage disequilibrium in finite populations. TAG Theoretical and Applied Genetics 38, 226-231.
