# Supplementary material for: Reconstructing Roma History from Genome-Wide Data
Source: PLoS One. 2013 Mar 13;8(3):e58633. doi: 10.1371/journal.pone.0058633 (PMC3596272; doi:10.1371/journal.pone.0058633)
Supplement: Note S2 — Simulations for estimating dates of admixture events. (PDF) [file pone.0058633.s014.pdf]

NOTE S2. Simulations for estimating dates of admixture events.

Simulation 1: To test the effect of founder events post admixture

In order to test the effect of founder events post admixture, we performed simulations using MaCS [1] coalescent simulator. We simulated data for three populations (say, *A*, *B* and *C*). We set the effective population size (N_e) for all populations to 12,500 (at all times except during the founder event), mutation and recombination rate were set $2x10^{-8}$ and to $1x10^{-8}$ per base pair per generation respectively. *C* can be considered as an admixed population that has 60%/40% ancestry from *A'* and *B'* (admixture time (*t*) was set to 30/ 100 generations before present). *A'* and *A* diverged 120 generations ago, *B'* and *B* diverged 200 generations ago and *A* and *B* diverged 1800 generations ago. At generation *x* ($x < t$), *C* undergoes a severe founder event where the effective population size (N_e) reduces to 5 individuals for one generation. At generation (*x+1*), the N_e = 12,500. We simulated data for 5 replicates for each parameter. We performed *ROLLOFF* analysis (using the original and modified statistic) with *C* as the target and *A* and *B* as the reference populations. When we use the original *ROLLOFF* statistic (*A(d)*), we observe that the dates are biased downward in cases of founder events post admixture. However, when we use the modified statistic (*R(d)*), the bias is removed (Table S3). Details of the bias correction are shown in Note S1. Throughout the manuscript, we use the modified *ROLLOFF* statistic (*R(d)*) unless specified otherwise.

Simulation 2: To test the accuracy of the modified *ROLLOFF* statistic (*R(d)*)

We perform simulations using the same simulation framework as in reference [2] to test the accuracy of the estimated dates using the modified *ROLLOFF* statistic. We simulated data for 25 admixed individuals using Europeans (HapMap CEU) and HGDP East Asians (Han) as ancestral populations, where mixture occurred between 10-300 generations ago and European ancestry proportion was set to

20%. These ancestral populations were chosen as F_{st}(CEU, Han) = 0.09 is similar to the F_{st} between the ancestral populations of the Roma (Europeans and ASI). Figure S4 shows that we get accurate estimates for the dates of mixture up to 300 generations.

Simulation 3: To test the effect of using PCA loadings instead of allele frequencies as weights in *ROLLOFF*

In the case of Roma admixture, data from unadmixed South Asian populations is not available and so it is not possible to compute the allele frequencies of SNPs for one of ancestral populations (ASI). However, data from many South Asian populations (which are admixed with ANI and ASI ancestry) are available and can be used for estimating the PCA-based SNP loadings. We performed simulations to mimic this scenario -

We simulated data for 60 admixed individuals using Europeans (HapMap CEU) and HGDP East Asians (Han) as ancestral populations, where mixture occurred 100 generations ago and European ancestry proportion was set to 30% (group 1: n = 20), 50% (group 2: n = 20) and 70% (group 3: n = 20). These three groups of simulated samples can be roughly considered as three South Asian populations. We performed PCA analysis with CEU and Groups 1-3 of simulated samples to estimate the SNP loadings that can be used in *ROLLOFF*.

Next, we simulated data for 54 individuals that can be used as the target in the *ROLLOFF* analysis. These individuals have 80%/20% European and East Asian ancestry respectively (similar to Roma) and the date of mixture is set to 30 (n = 27) and 100 (n = 27) generations before present. We ran *ROLLOFF* (using *R(d)*) to estimate the date of mixture in this panel of individuals using the PCA-based loadings computed above. We estimated that the dates of mixture were 33 ± 1 and 99 ± 1 generation for mixture that occurred 30 and 100 generations ago respectively (Figure S5). This shows that we can effectively estimate the date of

mixture even in the absence of data from unadmixed ancestral populations, as long as data from other admixed individuals (involving the relevant ancestral populations) is available.

Simulation 4: To test the model of two waves of admixture

In order to obtain an interpretation of the *ROLLOFF* estimated date of mixture when the assumption of single wave of mixture is incorrect, we ran *ROLLOFF* (using *R(d)*) to infer the date of admixture for data simulated under a two pulse admixture scenario. We simulated data using Europeans (HapMap CEU) and HGDP East Asians (Han) as the ancestral populations using the simulation framework described in reference [2]. We simulated two pulse admixture scenarios in which a 50%/50% admixture of CEU and Han occurred at λ_1, followed by a 60%/40% mixture of that admixed population and CEU at λ_2 (Table S4). The mixture proportions were chosen so that the final European ancestry proportion is ~80% (similar to Roma). We ran *ROLLOFF* (using *R(d)*) with a non-overlapping set of Europeans and Han as the reference population. Table S4 shows that as the interval between the time of the gene flow events (λ_2-λ_1) increases, the estimated dates of mixture reflects the date of the more recent gene flow event.

References

1. Chen GK, Marjoram P, Wall JD (2009) Fast and flexible simulation of DNA sequence data. Genome Research 19: 136-142.
2. Moorjani P, Patterson N, Hirschhorn JN, Keinan A, Hao L, et al. (2011) The History of African Gene Flow into Southern Europeans, Levantines, and Jews. PLoS Genetics 7: e1001373.
