# Supplementary material for: Reconstructing Roma History from Genome-Wide Data
Source: PLoS One. 2013 Mar 13;8(3):e58633. doi: 10.1371/journal.pone.0058633 (PMC3596272; doi:10.1371/journal.pone.0058633)
Supplement: Note S3 — Computing corrected IBD sharing distance between Roma and South Asian groups. (PDF) [file pone.0058633.s015.pdf]

NOTE S3. Computing corrected IBD sharing distance between Roma and South Asian groups.

To find the source of the South Asian ancestry in Roma, we inferred the pairwise IBD sharing distance between Roma and various South Asian groups using GERMLINE [1]. We observed that the Roma share the highest proportion of IBD sharing with groups from the northwest of India (Figure 3b). We were concerned that high IBD sharing could be an artifact related to the high proportion of ANI ancestry in the North-western Indian groups. Hence, we performed a regression analysis to correct for the effect of the ANI ancestry proportion on IBD sharing distance. The model that provided the best fit was IBD sharing = 0.35 + 0.81*ANI ancestry proportion (P-value < 0.05). Each South Asian group was considered as a single data point for this analysis. Next, we computed an average corrected IBD sharing measure for each region by regression out the effect of ANI ancestry and computing an average of the residuals for each region in India. Note: For this analysis, we did not include the Eastern Indian populations (Nysha and Ao Naga) and Andamanese populations (Onge and Great Andamanese) as these populations are not simple admixtures of ANI and ASI groups.

In order to control for the effect of the sample size on the IBD computation, we performed bootstrap analysis such that for each run, we randomly sampled up to 30 individuals (some groups had < 30 samples) from each of the 8 regional groups and estimated the IBD sharing statistics between Roma and the regional group. We performed a total of 100 runs and obtained the mean and standard error of the IBD statistic (Figure S7). We observed that Roma still share the highest proportion of IBD segments with groups from Northwest of India.

References

1. Gusev A, Lowe JK, Stoffel M, Daly MJ, Altshuler D, et al. (2009) Whole population, genome-wide mapping of hidden relatedness. Genome Research 19: 318.
