# Supplementary material for: Reconstructing Roma History from Genome-Wide Data
Source: PLoS One. 2013 Mar 13;8(3):e58633. doi: 10.1371/journal.pone.0058633 (PMC3596272; doi:10.1371/journal.pone.0058633)
Supplement: Note S4 — Simulations for estimating date of founder event. (PDF) [file pone.0058633.s016.pdf]

NOTE S4. Simulations for estimating date of founder event.

We used MaCS [1] coalescent simulator to perform simulations to test the robustness of our allele sharing statistic that we use for estimating the dates of the founder event. We simulate data for two populations (say, *A* and *B*) that diverged 1800 generations ago. We set the effective population size for both populations as N_e = 12,500, mutation rate = $2x10^{-8}$ and recombination rate = $1x10^{-8}$ per base pair per generation respectively. For each simulation, we compute the autocorrelation of allele sharing within individuals of *B*, and then subtract the cross-population autocorrelation between *A* and *B* to remove the effects of ancestral allele sharing (see Methods).

Simulation 1: Founder event only

B undergoes a severe founder event *x* generations ago where the effective population size reduces to 5 individuals for one generation. At generation (*x+1*), the population size = N_e again. Table S5 shows that in such cases we can accurately estimate the date of the founder event using our statistic.

Simulation 2: Founder event and admixture

We simulate data for a more complex demography where *B* is admixed and has 40% ancestry from *A'* which is closely related to *A*. The admixture occurred at time *t* and at time *x* = 10, 30 or 100 generations, *B* undergoes a severe founder event where the effective population size of *B* reduces to 5 individuals for one generation. Table S5 shows that for a recent founder event (10 and 30 generations ago), we accurately estimate the date of the founder event. However, for older founder events (100 generations), we are unable to accurately estimate the date of the founder event, if it occurred pre-admixture. However, this is expected as we are only sampling the admixed population (today) and not the ancestral population that underwent the founder event.

Simulation 3: No Founder event

We simulate data for a complex demography where *B* is admixed and has 40% ancestry from *A'* which is closely related to *A*. The admixture occurred 10, 30, 50 or 70 generations ago. In all cases, we observe that the allele-sharing statistic is not associated to distance. We test if the model of a straight line ($y \sim c$) or exponential decay ($y \sim c + Ae^{-tD}$), where *D* = genetic distance and *t* = time of founder event) provides a better fit to the output. In all four cases, we fail to reject the null model ($y \sim c$) (P > 0.05).

References

1. Chen GK, Marjoram P, Wall JD (2009) Fast and flexible simulation of DNA sequence data. Genome Research 19: 136-142.
